# MicroRNA-365 regulates human cardiac action potential duration

**DOI:** 10.1038/s41467-021-27856-7

**Published:** 2022-01-11

**Authors:** Dena Esfandyari, Bio Maria Ghéo Idrissou, Konstantin Hennis, Petros Avramopoulos, Anne Dueck, Ibrahim El-Battrawy, Laurenz Grüter, Melanie Annemarie Meier, Anna Christina Näger, Deepak Ramanujam, Tatjana Dorn, Thomas Meitinger, Christian Hagl, Hendrik Milting, Martin Borggrefe, Stefanie Fenske, Martin Biel, Andreas Dendorfer, Yassine Sassi, Alessandra Moretti, Stefan Engelhardt

**Affiliations:** 1 Institute of Pharmacology and Toxicology, Technical University of Munich (TUM), 80802 Munich, Germany; 2 DZHK (German Centre for Cardiovascular Research), partner site Munich Heart Alliance, 80802 Munich, Germany; 3 Center for Drug Research, Department of Pharmacy, Ludwig-Maximilians-Universität München, 81377 Munich, Germany; 4 First Department of Medicine, Faculty of Medicine, University Medical Centre Mannheim, 68167 Mannheim, Germany; 5 Klinik und Poliklinik für Innere Medizin I, Klinikum rechts der Isar, Technical University of Munich (TUM), 81675 Munich, Germany; 6 Institute of Human Genetics, Klinikum rechts der Isar, Technical University of Munich (TUM), 81675 Munich, Germany; 7 Clinic of Cardiac Surgery, University Hospital, Ludwig-Maximilians-Universität München, 81377 Munich, Germany; 8 Erich and Hanna Klessmann Institute, Heart and Diabetes Center NRW, University Hospital of the Ruhr-University Bochum, Bad Oeynhausen, Germany; 9 Walter-Brendel-Centre of Experimental Medicine, University Hospital, Ludwig-Maximilians-Universität München, 81377 Munich, Germany; 10 Present Address: Fralin Biomedical Research Institute at Virginia Tech Carilion, Roanoke, VA USA

**Keywords:** Nucleic-acid therapeutics, miRNAs, Arrhythmias

## Abstract

Abnormalities of ventricular action potential cause malignant cardiac arrhythmias and sudden cardiac death. Here, we aim to identify microRNAs that regulate the human cardiac action potential and ask whether their manipulation allows for therapeutic modulation of action potential abnormalities. Quantitative analysis of the microRNA targetomes in human cardiac myocytes identifies miR-365 as a primary microRNA to regulate repolarizing ion channels. Action potential recordings in patient-specific induced pluripotent stem cell-derived cardiac myocytes show that elevation of miR-365 significantly prolongs action potential duration in myocytes derived from a Short-QT syndrome patient, whereas specific inhibition of miR-365 normalizes pathologically prolonged action potential in Long-QT syndrome myocytes. Transcriptome analyses in these cells at bulk and single-cell level corroborate the key cardiac repolarizing channels as direct targets of miR-365, together with functionally synergistic regulation of additional action potential-regulating genes by this microRNA. Whole-cell patch-clamp experiments confirm miR-365-dependent regulation of repolarizing ionic current I_ks_. Finally, refractory period measurements in human myocardial slices substantiate the regulatory effect of miR-365 on action potential in adult human myocardial tissue. Our results delineate miR-365 to regulate human cardiac action potential duration by targeting key factors of cardiac repolarization.

## Abstract (web summary)

An abnormal cardiac action potential underlies different types of cardiac arrhythmias. Here the authors show that microRNA-365 regulates the cardiac action potential by modulating key cardiac repolarizing channels.

## Introduction

The generation of physiological cardiac action potential (AP) belongs to the most tightly controlled physiological processes of the human body. A sophisticated timed series of activation and deactivation (sequential gating) of cardiac ion channels guarantees the extraordinary robustness of this process^1,2^. Reduction of repolarizing currents, typically due to heterozygous mutations in ion channel genes, can shift the balance of currents toward prolongation of AP duration and QT interval, known as Long-QT syndrome (LQTS), while an increase of outward currents causes shortening of repolarization duration and thus shorter QT intervals (Short-QT syndrome, SQTS). Such ventricular arrhythmias may progress to ventricular fibrillation and sudden cardiac death (SCD)^3–5^.

A large number of disease-causing mutations in cardiac ion channels, their accessory subunits or regulators have been reported, with the majority being heterozygous^6^. Both the mutant and healthy allele remain subject to post-transcriptional regulation, which could be a target for therapeutic intervention. MicroRNAs (miRNAs) and their manipulation provide such a therapeutic entry point, as they post-transcriptionally regulate a large fraction of mammalian mRNAs^7^, including those encoding for many cardiac ion channels.

A regulatory role of distinct miRNAs has been reported for various aspects of cardiac rhythm control and arrhythmias^8^. However, the majority of these miRNAs target processes that affect cardiac rhythm control beyond the direct targeting of cardiac myocyte AP-determining ion channel mRNAs. Examples for these are miR-21 and miR-26, promoting atrial fibrillation through their pro-fibrotic role and disrupting Ca^2+^ signaling in fibroblasts, respectively (reviewed in ref. ^8^). In contrast, only a few miRNAs, namely cardiac miRNAs miR-1^9^ and miR-133, as well as miR-19^10^ have been shown to regulate ventricular repolarization through direct targeting of the relevant ion channels in rodent and zebrafish models, respectively (reviewed in ref. ^11^).

Here, we sought to systematically identify those miRNAs that through their sequence-specific targetome exert the highest potential as to the control of human cardiac AP repolarization. This resulted in the identification of miR-365, which we report to directly regulate the functionally dominating repolarizing ion channels in the human heart. Molecular characterization and manipulation of miR-365 in human cardiac models suggest miR-365 as an important regulator of human cardiac AP duration.

## Results

### MiR-365 is a primary miRNA controlling cardiac repolarizing ion channels in human myocytes

**Fig. 1:**
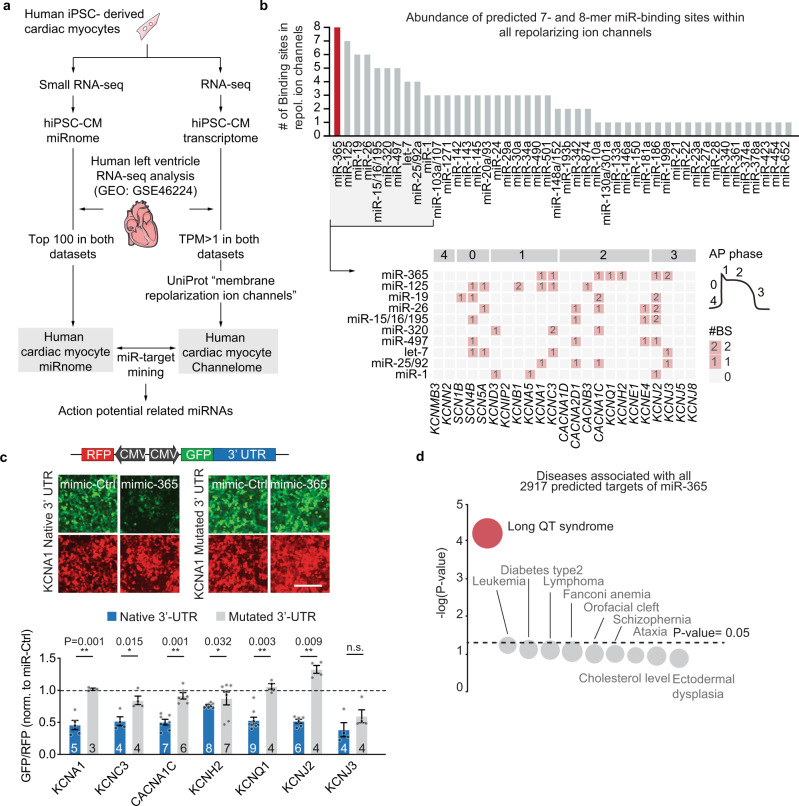
The targetome of miR-365 in the human myocardium links this miRNA to cardiac repolarization and arrhythmia. **a** Screening of miRNA targetomes to identify miRNAs involved in the regulation of cardiac repolarization (The scheme was prepared using Servier Medical Art). **b** Number of the predicted binding sites within 3’ UTRs of mRNAs encoding for repolarizing ion channels. MiRNAs are sorted according to the respective number of binding sites (upper panel). The ion channels targeted by the top 10 candidate miRNAs and their assignment to the different phases of ventricular action potential (AP, lower panel). The analysis involved all of the miRNAs and repolarizing ion channels that are expressed in the human myocardium. **c** Reporter assay to test for direct interaction between miR-365 and its predicted ion channel targets. HEK293 cells were transfected with double fluorescent reporter constructs carrying 3’-UTR sequences of target genes, including native or mutated binding sites for miR-365. Representative images of cells expressing a reporter with native or mutated *KCNA1* 3’ UTR and transfected with mimic-365 or -Ctrl (scale bar = 25 µm, upper panel). The lower panel depicts the quantitative analysis of the results. Data are presented as mean ± SEM and acquired from 3–9 independent experiments, each performed in triplicate. Source data and detailed statistical analyses are provided as a Source Data file. **d** Bubble plot depicting the outcome of gene list enrichment analysis to evaluate the association between all the predicted targets of miR-365 (2917 transcripts) and all hereditary human diseases listed in the OMIM database. Bubble size corresponds to the contribution of target genes to each disease term. The top 10 associated diseases are shown. The *y*-axis depicts the *p*-values calculated using the Fisher exact test. *P*-value < 0.05 was considered significant.

In an effort to identify miRNAs that control the human cardiac AP, we aimed for quantitative analysis of miRNA targetomes in human cardiac myocytes (Fig. 1a). To this end, deep RNA sequencing and small RNA sequencing of human cardiac myocytes derived from healthy induced pluripotent stem cells (hiPSC-CMs) were complemented by transcriptome and small RNA profiling datasets of human non-failing myocardium^12^ (GEO accession: GSE46224). To assess the human cardiac channelome in these data, we first extracted a list of 27 genes from the UniProt Knowledge-based database^13^, which code for proteins that are confidently annotated and reviewed as ion channels involved in the repolarization phase of the AP. Out of these 27 ion channels, 23 were robustly expressed in cardiac myocytes, based on the expression profiles of adult human myocardium and hiPSC-CMs. With regard to miRNA stratification, we then considered miRNAs beyond a common threshold of expression in both adult human myocardium and hiPSC-CMs (top 100 highly expressed miRNAs) for further downstream analysis, as cellular miRNA concentration is closely linked to its functional relevance^7^. MiRNA-target mining using TargetScan v7.2^14^ yielded 273 individual miRNAs (46 seed families beyond the expression threshold) with corresponding conserved and canonical 7- or 8-mer binding sites within the 3’ untranslated regions (UTRs) of cardiac ion channel genes (Fig. 1b). Among these top cardiac ion channel-targeting miRNAs, miR-365 exhibits the largest number (8) of binding sites in the 3’ UTR of cardiac ion channel mRNAs, followed by miR-125 (7 binding sites) and miR-26 (6 binding sites). Intriguingly, the predicted direct targets of miR-365 (i.e., *KCNQ1*, *KCNH2*, *KCNJ2*, *CACNA1C*, *KCNC3*, *KCNA1*, and *KCNJ3*, Supplementary Fig. 1a) are known as the dominating ion channel isoforms that determine cardiac repolarization^1^ (AP phases 1, 2, and 3, Fig. 1b, lower panel). In order to validate the direct interaction between miR-365 and the 3’ UTR of these predicted ion channel targets, we carried out double-fluorescent reporter assays in HEK293 cells. We used reporter constructs that express RFP and GFP under control of identical but independent promoters, with RFP serving as an internal control, while GFP is followed by the native 3’ UTR of the target gene or a seed-mutated 3’ UTR as control. Fluorescent intensities were then assessed by high content microscopy at the single-cell level and the GFP/RFP ratio served as a measure of miRNA binding and repression of the specific target 3’ UTR. Augmentation of miR-365 using a synthetic miR-365 mimic (mimic-365) lowered the GFP/RFP ratio compared to scrambled control (mimic-Ctrl), showing the capability of miR-365 to interact with its binding sites within the 3’ UTR of the predicted conserved targets and repress these ion channels mRNAs (Fig. 1c, blue bars). The effect was not observed in mutated constructs showing the specificity of miRNA-binding site interaction (Fig. 1c, gray bars).

As an independent approach to predict the functionality of miR-365, we performed gene enrichment analysis for predicted targets of miR-365 in human hereditary disease phenotypes. This primarily associated the targetome of miR-365 to LQTS among all genetic diseases in humans (OMIM database), suggesting a potential role of miR-365 in controlling the AP and arrhythmogenesis (Fig. d).

As the expression level of ion channels that determine the cardiac AP and also the expression of miR-365 exhibit a high degree of species specificity^15^ (Supplementary Fig. 1b, c), we sought to study this miRNA in human cardiac cells and models.

### MiR-365 levels determine AP duration in human iPSC-derived cardiac myocytes

**Fig. 2:**
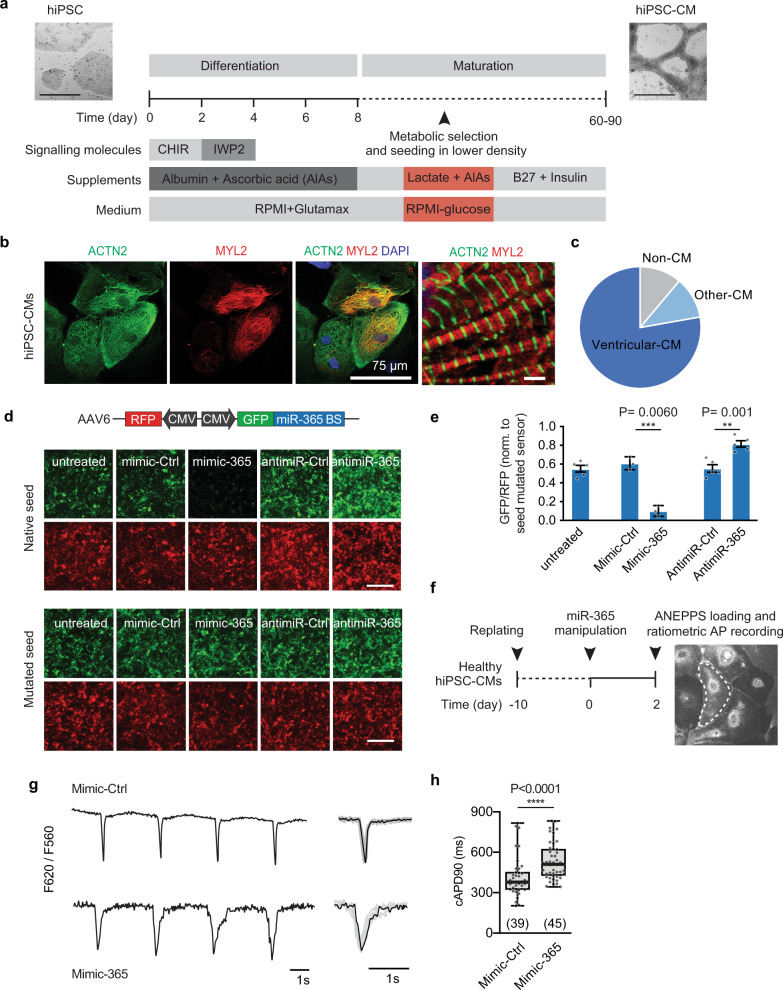
MiR-365 prolongs action potential duration in human iPSC-derived cardiac myocytes. **a** Protocol for differentiation of the patient-specific iPSCs (left, hiPSC) to cardiac myocytes (right, hiPSC-CMs). The scale bars represent 500 μm. **b** The structural organization of the myofilaments in hiPSC-CMs, assessed by immunofluorescent staining for actinin and myosin filaments (large scale bar = 25 μm, small scale bar = 2 μm). **c** The proportion of cell types at day 60 of cardiac differentiation. Myocytes were defined as cells staining positive for alpha-actinin (ACTN2). Ventricular-like CMs expressed the ventricular marker myosin light chain 2 (MYL2) in addition to alpha-actinin (Data from 3 independent differentiations). **d** Double fluorescent sensor for miR-365 activity in living cells. Representative images of hiPSC-CMs infected with AAV6 expressing miR-365 activity sensor with a native or mutated seed sequence. The activity of miR-365 was determined by high content microscopy upon transfection with mimics or antimiRs (scale bar = 100 μm). **e** Quantification of data from **d**. Data are from 3–5 independent experiments performed in duplicates presented as mean ± SEM. **f** Scheme showing optical AP recordings using di-8-ANEPPS upon manipulation of miR-365 levels in hiPSC-CMs, done using MetaFluor software. **g** Representative action potential traces (F655/F560) from healthy hiPSC-CMs transfected with mimic-365 or control mimic and were loaded 48 h later with di-8-ANEPPS (left). Average trace of 15 APs recorded from a representative cell in each condition (right). **h** Quantitative analysis of the ratiometric determination of transmembrane potential (median 379 ms in the control group compared to 509 ms in the mimic-365 group). All measurements were performed at 35 °C. Data are represented as Bazett’s corrected action potential duration at 90% of the repolarization (cAPD90). Data are acquired from 4 independent experiments and presented as Box (25% and 75% quartiles) and whisker (min to max) showing the median and individual values. Source data and detailed statistical analyses for **c**, **e** and **h** are provided as a Source Data file.

As a model for human cardiac myocytes, we used hiPSCs from a healthy individual (i.e., no history of cardiovascular disease)^16^ and applied an established cardiac differentiation protocol to obtain beating hiPSC-CMs^17^ with minor modifications (Fig. 2a). Staining against ACTN2 (Actinin Alpha 2), a general cardiac myocyte marker, and a ventricular specific marker, MYL2 (ventricular myosin light chain 2), revealed that the differentiation resulted in a heterogeneous population consisting of a majority of ventricular-like myocytes (ACTN2^+^MYL2^+^), as well as a population of non-ventricular myocytes (ACTN2^+^MYL2^−^) and a smaller fraction of other cells lacking both markers (Fig. 2b, c).

To assess the activity of miR-365 within hiPSC-CMs and upon its manipulation, we generated double-fluorescent reporters carrying native or seed-mutated binding sites (BS) for miR-365 downstream of the cDNA of GFP. We employed adeno-associated virus serotype 6 (AAV6) to express this construct robustly in hiPSC-CMs (Fig. 2d). We then assessed the GFP/RFP ratio as a measure for miRNA presence and activity by high content microscopy, and we found 25 nM of synthetic mimic- or antimiR-365 to exert significant and robust elevation or inhibition of miRNA activity, respectively (Fig. 2d, e).

To determine the regulatory impact of miR-365 on the cardiac AP, we measured AP duration in hiPSC-CMs upon transfection with mimic-365. We used a voltage-sensitive dye, di-8-ANEPPS, to perform high throughput, single-cell optical recordings of the AP in spontaneously contracting hiPSC-CMs and reported this as beating frequency-corrected AP duration at 90% of repolarization (cAPD90, Fig. 2f). Mimic-365 significantly increased AP duration compared to mimic-Ctrl-treated cells (Fig. 2g, h). Similar results were obtained by multielectrode array (MEA) experiments in neonatal rat cardiac myocytes (Supplementary Fig. d, e).

### Modulation of miR-365 in human models of QT syndromes

**Fig. 3:**
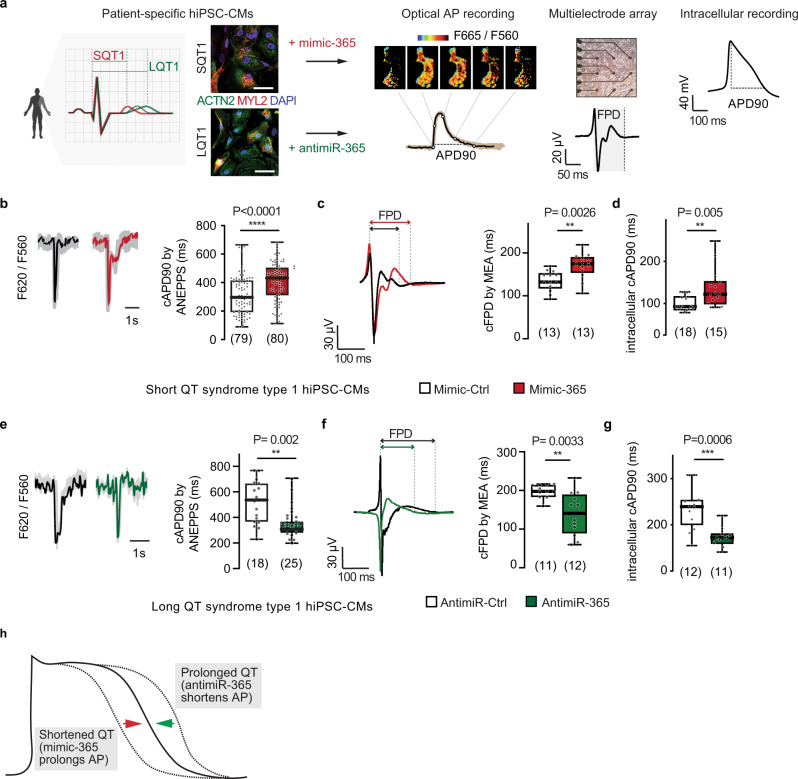
Modulation of miR-365 in human models of QT syndromes. **a** Experimental design for AP recordings in patient-specific SQT1 and LQT1 hiPSC-CMs upon manipulation of miR-365 (scheme prepared using BioRender.). Representative immunostainings of SQT1 and LQT1 hiPSC-CMs (>60 days) showing the expression of cardiac markers ACTN2 and MYL2 (scale bar = 25 μm). **b** Average of 15 representative AP traces (F655/F560) from SQT1 hiPSC-CMs that were transfected with mimic-365 (red) or mimic-Ctrl (black) and were loaded 48 h later with di-8-ANEPPS (left panel). Quantification of optical AP recordings in SQT1 hiPSC-CMs (right panel). Median beating rate-corrected APDs at 90% of repolarization (cAPD90) were 290 ms in mimic-Ctrl and 431 ms in mimic-365-treated cells (data from 3 independent experiments). **c** Representative field potential (FP) traces recorded from SQT1 hiPSC-CMs upon treatment with mimic-365 (red) or -Ctrl (black) using a multielectrode array (MEA) system (left panel). Quantification of FP duration corrected for beating frequency (cFPD, right panel). **d** cAPD90 measured from SQT1 hiPSC-CMs upon manipulation of miR-365 using sharp microelectrodes for intracellular AP recording. **e** Averaged F665/F560 traces from 5 to 10 recorded APs (left panel) in representative LQT1 hiPSC-CMs treated with either antimiR-Ctrl (black) or antimiR-365 (green). Quantification of optical AP recordings in the LQT1 hiPSC-CMs (right panel). Data are from 3 independent experiments. Median cAPD90 in control- and antimiR-365 treated cells were 534 ms and 401 ms, respectively. **f** Representative FP traces of LQT1 hiPSC-CMs transfected with antimiR-365 or -Ctrl (left panel) and quantification of cFPD recorded post-transfection. **g** Intracellular AP recordings from LQT1 hiPSC-CMs treated with antimiR-Ctrl or -365 using sharp microelectrodes. **h** Scheme representing the effects of miR-365 manipulation on AP duration in QT syndromes. All quantitative data are plotted as box (25% and 75% quartiles) and whisker (min to max) showing the median and individual values. Source data and detailed statistical analyses for (**b**–**g**) are provided as a Source Data file.

We next sought to manipulate miR-365 in human cardiac myocytes with pathologically altered AP duration and used hiPSC-CM from patients suffering from Long-QT syndrome type 1 (LQT1)^18^ and Short-QT syndrome type 1 (SQT1)^19^, respectively. Employing optimized cardiac differentiation conditions^17^, we achieved a high fraction of ventricular myocytes (ACTN2 and MYL2-positive) after 60 days for both LQT1 and SQT1 (Fig. 3a). We have shown previously that the altered QT interval measured by electrocardiography in the donor patients is reflected in the AP duration recorded from the respective patient-derived hiPSC-CMs^18,19^. Therefore, we aimed to determine the AP duration in these cells upon manipulation of miR-365 by measuring changes in membrane voltage using the voltage-sensitive dye di-8-ANEPPS, field potential duration (FPD) using a MEA system, and intracellular AP using sharp microelectrodes (Fig. 3a).

Based on our observation that augmented levels of miR-365 prolonged the AP duration in healthy cells, we treated SQT1 hiPSC-CMs with mimic-365. Optical AP recordings revealed that this treatment resulted in about 30% of prolongation in the pathologically shortened AP duration (median 290–431 ms, Fig. 3b). Similarly, the beating frequency-corrected FPD (cFPD) was prolonged by 58 ± 18 ms in SQT1 hiPSC-CMs treated with mimic-365 compared to the cells transfected with mimic-Ctrl (Fig. 3c). In addition, we performed intracellular AP recordings in spontaneously beating SQT1 hiPSC-CMs using sharp microelectrodes. These recordings substantiated our observation showing a 24% increase in AP duration upon miR-365 overexpression (Fig. 3d). As our cardiac differentiation protocol yields a heterogeneous cardiac myocyte population, we additionally sought to record AP duration exclusively from the ventricular subpopulation within the CMs. To this end, we transduced hiPSC-CMs with a recombinant AAV (AAV6-MYL2-VSFP-CR) that encodes a fluorescent resonance energy transfer (FRET)-based voltage sensor, which is under control of the ventricular myocyte-specific MYL2 promoter^20^ (Supplementary Fig. 2a, b). These measurements showed likewise a normalization of AP duration upon overexpression of miR-365 in SQT1 hiPSC-CMs (Supplementary Fig. 2c-e).

In LQT1 hiPSC-CMs, we employed a synthetic, locked nucleic acid (LNA)-modified oligonucleotide inhibitor of miR-365 (antimiR-365) or a scrambled oligonucleotide as a negative control (antimiR-Ctrl). Optical AP duration measurements showed a 40% reduction of cAPD90 upon antimiR-365 transfection (median 346 ms compared to 534 ms in antimiR-Ctrl treated cells, Fig. 3e). This observation was in agreement with MEA measurements, where administration of antimiR-365 resulted in 59 ± 18 ms shortening of cFPD in LQT1 hiPSC-CMs (Fig. 3f). Similarly, intracellular AP recordings revealed 26% reduction in cAPD90 upon antimiR-365 transfection (median 172 ms) compared to antimiR-Ctrl group (median 239 ms, Fig. 3g). FRET-based recordings in the ventricular population corroborated a shortening and thus normalization of the abnormally prolonged AP to comparably normal levels (median 673 ms in the antimiR-Ctrl group compared to 418 ms in antimiR-365 treated cells; Supplementary Fig. 2f-h).

Taken together, the results obtained in myocytes derived from patients with abnormal AP duration provided proof of concept for the adjustment of AP duration by manipulation of miR-365 activity through synthetic oligonucleotides (Fig. 3h).

### MiR-365 regulates key repolarizing ion channels in ventricular myocytes

**Fig. 4:**
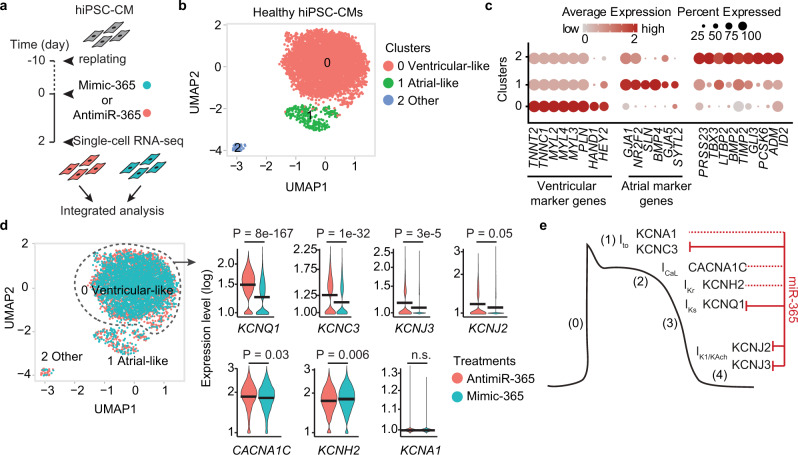
MiR-365 regulates the key repolarizing potassium channels in ventricular myocytes. **a** Schematic overview of the single-cell RNA-sequencing experiment in healthy hiPSC-CMs treated with mimic- or antimiR-365. **b** Uniform manifold approximation and projection (UMAP) plot representing the unbiased clustering of 6000 transcriptomes revealed three main populations of cells, namely ventricular-like, atrial-like, and other cardiac myocytes. **c** Dot plot showing the expression of marker genes defining each subpopulation of hiPSC-CMs. **d** Combined UMAP clustering of hiPSC-CM treated with mimic- or antimiR-365 to depict the contribution of cells from each sample to different hiPSC-CM clusters (left panel). Violin plots representing the expression changes of ion channel targets of miR-365 in ventricular-like cardiac myocytes upon manipulation of miR-365 (right panel). Analyses were performed using CellRanger and Seurat (v3.2) packages. Adjusted *P*-value was calculated using two-sided Wilcoxon Rank Sum test for each gene. **e** Contribution of the validated targets of miR-365 in different phases of ventricular myocyte AP. Solid lines represent miR-365-based regulation at the transcriptome level, while the dotted lines depict a potential interaction between miR-365 and the ion channel mRNAs without a relevant change at mRNA level.

Due to the fact that current hiPSC-CM differentiation protocols, typically yield a heterogeneous population of cardiac myocytes, we aimed to delineate the composition of the hiPSC-CM population and assess the impact of miR-365 manipulation in different fractions of hiPSC-CMs. We performed single-cell RNA sequencing (scRNA-seq) of healthy hiPSC-CMs upon transfection with mimic- or antimiR-365 (Fig. 4a, Supplementary Fig. 3a-c) and combined the two resulting datasets into a Uniform Manifold Approximation and Projection (UMAP) embedding. Unsupervised clustering of 5200 transcriptomes revealed three main populations of cells (Fig. 4b) and using transcriptomic maps of cardiogenesis^21,22^ we assigned cardiac myocyte types to these clusters. All cells expressed general cardiac markers, such as various types of Troponin and Myosin, at different expression levels. The majority of cells (about 90%) showed high levels of *MYL2* as well as expression of ventricular myocyte-specific genes such as *PLN*, *HAND1,* and *HEY2*, and were categorized as ventricular-like cardiac myocytes. Atrial-like myocytes (8% of the population) followed, with an exclusive expression of *GJA5*, *NR2F2*, *BMP4,* and *SLN* and a small population of myocytes (about 2%) expressing several markers genes found in outflow-tract (OFT)-like myocytes (e.g., *BMP2*, *TBX3*, *PRSS23*, *ID2*, *ADM,* and *ITM2A*) (Fig. 4c). Manipulation of miR-365 did not alter the cell composition, with three populations present in both samples (Fig. 4d, Supplementary Fig. 3d).

We then determined the expression of repolarization-associated ion channels we had identified as potential targets of miR-365 in our initial bioinformatic prediction (Fig. 4d). Among these, *KCNQ1, KCNC3, KCNJ3,* and *KCNJ2* were robustly and significantly repressed in mimic-365-treated cells. In contrast, the AP-prolonging *CACNA1C* showed only subtle repression. Although we showed in HEK293 cells that miR-365 can interact with and repress *KCNH2*, we did not find significant regulation upon manipulation of endogenous miR-365 in hiPSC-CM, suggesting that additional regulatory mechanisms compensate for and overrule the effect of miR-365 on this target. Among the predicted ion channel targets of miR-365, *KCNA1* was hardly detected in the ventricular myocytes.

A similar pattern of target repression was observed based on RNA-seq experiments in SQT1 hiPSC-CM treated with mimic-365 compared to mimic-Ctrl (Supplementary Fig. 4a-c), with the exception of *KCNJ2*, which showed lower expression compared to healthy cells already under control conditions and the slight repression by miR-365 did not reach statistical significance.

In addition, we performed scRNA-seq in LQT1 hiPSC-CMs upon inhibition of miR-365 (Supplementary Fig. 5a), where we observed ventricular-, atrial-, and OFT-like myocyte populations (Supplementary Fig. 5b-d). We found *KCNQ1*, *KCNH2*, *KCNC3,* and *KCNJ3* to be significantly derepressed upon inhibition of miR-365, whereas *CACNA1C* showed unchanged levels compared to untreated control. The expression level of *KCNJ2* and *KCNA1* in these cells were below the level for reliable detection of differential expression.

Taken together, these data identify miR-365 to target and repress some of the most important repolarizing ion channels in human ventricular myocytes (Fig. 4e).

### MiR-365 shapes a transcriptome signature that prolongs repolarization beyond repressing its direct ion channel targets

**Fig. 5:**
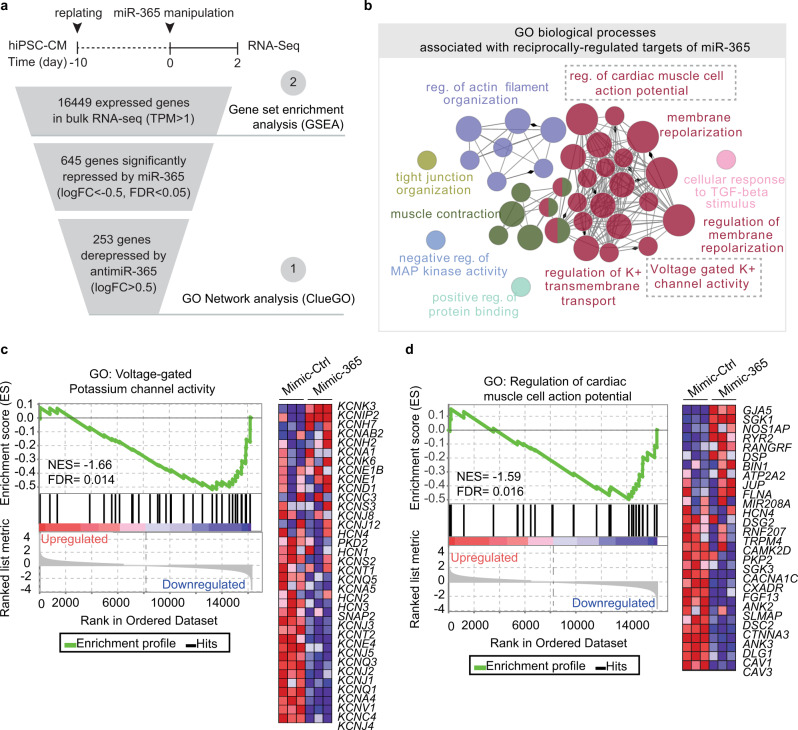
MiR-365 shapes the human cardiac AP by repressing the AP-regulatory network. **a** RNA-seq experimental scheme and filtering steps for shortlisting the expressed genes and the differentially expressed genes for downstream gene set enrichment analysis (2) and GO term identification (1), respectively. **b** Functionally grouped gene ontology network of biological processes significantly associated with miR-365 target genes. Each bubble (network node) represents a specific biological process or pathway, and lines (network edges) show the relations between different terms. Nodes of the same color belong to functionally similar terms or pathways. The node size represents the term enrichment significance (*P*-value < 0.0005 to <0.05). **c**, **d** Testing of regulation of the GO terms voltage-gated potassium channel activity (**c**) and regulation of cardiac muscle cell action potential (**d**) by gene set enrichment analysis (GSEA) upon comparing the transcriptomes of hiPSC-CMs treated with mimic-365 or mimic-Ctrl Genes comprising each GO term are shown in the heatmaps were a spectrum of blue to red depicts low to high expression of each gene. Normalized enrichment score (NES) determines whether the term is negatively or positively enriched upon miR-365 elevation. A false discovery rate (FDR) of less than 0.05 is considered as significant enrichment.

Next, we complemented our scRNA-seq experiments on miR-365-manipulated hiPSC-CMs with deep RNA sequencing for better coverage of lowly expressed genes (Fig. 5a, Supplementary Fig. 6a, b). Using this approach, we identified about 16,000 expressed genes (transcripts per million reads, TPM > 1, compared to about 4000 in scRNA-seq), among which 645 genes were significantly downregulated in hiPSC-CMs treated with mimic-365 compared to mimic-Ctrl. Out of these repressed genes, 253 were reciprocally regulated (derepressed) in antimiR-365-treated hiPSC-CMs (Fig. 5a). To assess whether these transcriptome changes are indeed related to the modulation of miR-365 activity, we determined the distribution of mRNA-fold changes for all genes and compared that to those of mRNAs predicted to contain 7- or 8-mer binding sites for miR-365 (Supplementary Fig. 6c, d). Elevation of miR-365 resulted in a significant reduction of predicted target mRNAs, whereas inhibition of miR-365 significantly derepressed miR-365 targets compared to mRNAs containing no binding site for miR-365 (Supplementary Fig. 6c, d). To determine the biological processes primarily affected by miR-365 deregulation, we next conducted gene ontology (GO) enrichment analysis on the 253 genes reciprocally regulated upon elevation and inhibition of miR-365, respectively. Among all biological processes known in humans, voltage-gated potassium ion transport and regulation of cardiac AP were highly enriched (Fig. 5b, depicted in red). To assess the effect of miR-365 elevation on the expression pattern of each component of these gene ontologies, we then performed gene set enrichment analysis (GSEA). GSEA yielded a significantly negative normalized enrichment score (NES) for the terms “voltage-gated potassium channel activity” (NES = −1.66, FDR < 0.014, Fig. 5c) and “regulation of cardiac muscle cell action potential” (NES = −1.59, FDR < 0.016, Fig. 5d). This analysis corroborated the scRNA-seq results on the repression of the predicted direct targets of miR-365 (*KCNQ1*, *KCNJ2*, and *KCNJ3*), but also revealed downregulation of other ion channel pore-forming or accessory genes and important regulatory factors such as caveolins (*CAV1* and *CAV3*), Ankyrins (*ANK3* and *ANK2*) and potassium channel-regulating kinases (e.g., *SGK3*; Fig. 5c, d; heatmaps). Except for *ANK3* (which contains a predicted 7-mer binding site for miR-365), the transcripts of these genes do not carry canonical binding sites for miR-365, indicating that miR-365 shapes a transcriptome signature in favor of AP prolongation beyond repressing its direct ion channel targets. This set of AP-modulatory genes regulated by miR-365 was also found to respond to manipulation of miR-365 in LQT1 hiPSC-CMs corroborating the findings in hiPSC-CM from healthy individuals (Supplementary Fig. 5f, g).

### Electrophysiological characterization of miR-365-targeted ionic currents in human models of QT syndromes

**Fig. 6:**
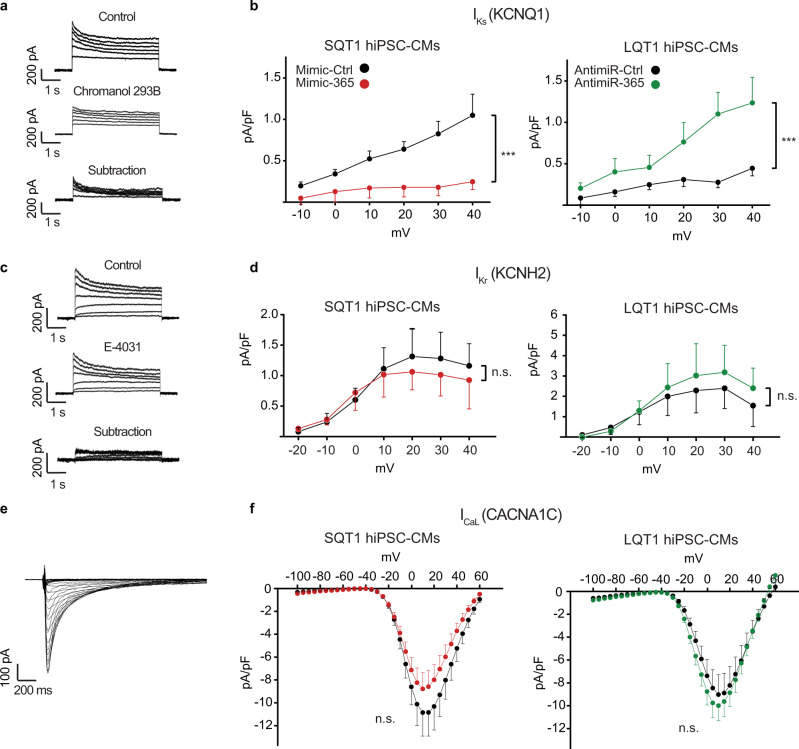
Electrophysiological characterization of miR-365-targeted ionic currents in human models of QT syndromes. **a** Representative recordings of the slow delayed rectifier potassium current (I_Ks_, pore-forming subunit: KCNQ1) in SQT1 hiPSC-CMs. Upper, middle and lower traces depict control, chromanol 293B (10 µM), and chromanol 293B-subtraction, respectively. **b** Current-voltage relationship (IV curve) of I_Ks_ in SQT1 hiPSC-CMs (left panel) upon treatment with mimic-365 (*n* = 15, red) or mimic-Ctrl (*n* = 12, black) and in LQT1 hiPSC-CMs (right panel) upon transfection with antimiR-365 (*n* = 10, green) or antimiR-Ctrl (*n* = 13, black). **c** Representative recordings of the rapid delayed rectifier potassium current (I_Kr_, pore-forming subunit: KCNH2) in LQT1 hiPSC-CMs. Upper, middle, and lower traces represent control, E-4031 (1 µM), and E-4031-subtraction, respectively. **d** Current-voltage relationship of I_Kr_ in SQT1 (left panel) and LQT1 hiPSC-CMs (right panel) upon manipulation of miR-365 (*n* = 11 and 8 cells in SQT1 cells treated with mimic-365 and mimic-Ctrl, and 10 and 7 cells in antimiR-365 and antimiR-Ctrl-treated LQT1 hiPSC-CMs). **e** Representative I_CaL_ recording (pore-forming subunit: CACNA1C) in an SQT1 hiPSC-CM. **f** Current-voltage relationship of I_CaL_ in SQT1 hiPSC-CMs (left panel) after treatment with mimic-365 (*n* = 14) or mimic-Ctrl (*n* = 14). IV curve of I_CaL_ in LQT1 hiPSC-CMs (right panel) after treatment with antimiR-365 (*n* = 12) or antimiR-Ctrl (*n* = 8). Error bars represent the standard error of the mean (SEM). Statistical significance was tested with two-way ANOVA for repeated measures followed by Sidakholm post-hoc analysis (***P-value < 0.001; n.s., not significant). Source data are provided as a Source Data file.

To directly measure the main ionic currents deriving the miR-365-dependent regulation of AP duration in hiPSC-CMs, we employed the whole-cell patch-clamp technique. Delayed rectifying potassium currents I_Ks_ and I_Kr_ (pore-forming subunits encoded by *KCNQ1* and *KCNH2*, respectively) and the L-type calcium current I_CaL_ (pore-forming subunit encoded by *CACNA1C*) were recorded upon manipulation of miR-365 in LQT1 and SQT1 hiPSC-CMs. I_Ks_ was measured as chromanol 293B-sensitive outward potassium current (Fig. 6a). These measurements showed a significant reduction of I_Ks_ in SQT1 hiPSC-CMs upon treatment with mimic-365 and significant augmentation of I_Ks_ in LQT1 hiPSC-CMs treated with antimiR-365, compared to the respective controls (Fig. 6b). These results are in line with the regulation of *KCNQ1* mRNA by miR-365 (Fig. 4d) and confirm the prominent contribution of I_Ks_ to the observed effects of miR-365 on human AP duration. I_Kr_, measured as E-4031-sensitive outward potassium current (Fig. 6c) showed smaller changes in current densities upon manipulation of miR-365, which did not reach statistical significance (Fig. 6d). There were no significant changes in current densities of I_CaL_ upon treatment with mimic-365 or antimiR-365 (Fig. 6e, f), in line with the subtle changes at the mRNA level (Fig. 4d). The inwardly rectifying potassium current I_K1_ (main subunit encoded by *KCNJ2*), another key determinant of cardiac repolarization, was found to be extremely small in both SQT1 and LQT1 hiPSC-CMs in our setting. This precluded us from determining miR-365-dependent changes of I_K1_ in hiPSC-CMs.

Collectively, our electrophysiological measurements in hiPSC-CMs revealed a major contribution of I_Ks_ regulation to the overall effect of miR-365 on AP duration in human myocytes.

### Functional characterization of miR-365-based action potential regulation in human myocardium

**Fig. 7:**
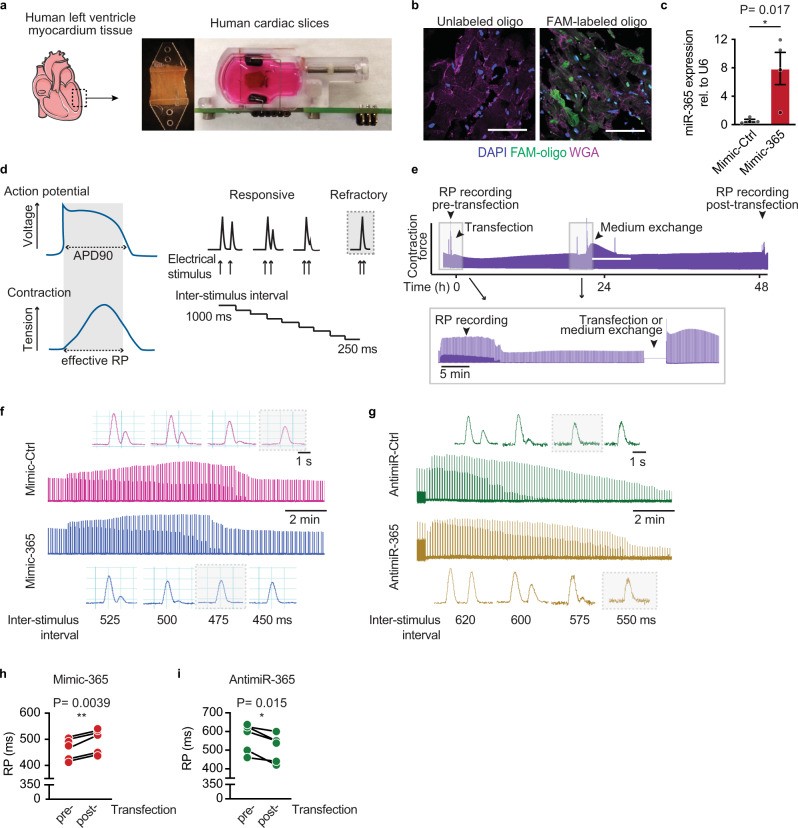
Functional characterization of miR-365-based action potential regulation in human myocardium. **a** Slices were prepared from human ventricular myocardium with 300 µm thickness, glued to plastic triangles and mounted in biomimetic tissue culture chambers that enable constant measurement of contraction force. The slices were maintained under 1 mN preload and 0.5 Hz electrical pacing for at least two weeks before starting the functional experiments to reach an equilibrium (scheme created using Servier Medical Art). **b** Confocal microscopy images of 5 µm sections prepared from myocardial slices transfected with Fluorescein amidite (FAM)-labeled oligonucleotides (green) compared to an untreated slice. Wheat germ agglutinin staining (WGA, magenta) defines the area of the cells and DAPI (blue) marks the nuclei (scale bar = 75 µm). **c** MiR-365 levels in mimic-treated slices as determined by qRT-PCR. Data are presented as mean ± SEM (*n* = 4 independent samples). **d** The correlation between the effective refractory period and AP duration in ventricular myocytes (left panel) and the automated electrical stimulation protocol for determining the RP. The point where the myocardial slice reaches refractoriness is marked with a grey dashed frame (right panel). **e** Experimental scheme representing the timeline for transfection of cardiac slices with synthetic oligonucleotides following by medium exchange after 24 h. RP measurements were performed before and 48 h after miR-365 manipulation. **f** Representative RP measurement traces from slices treated with mimic-Ctrl (pink) or mimic-365 (blue) 48 h after the transfection. Grey dashed frames in high-resolution traces depict the refractoriness of each slice. **g** Representative contraction force traces acquired from slices transfected with antimiR-Ctrl (green) or antimiR-365 (brown) 48 h after transfection. The refractoriness of slices is marked with a gray dashed frame. **h**, **i** Quantitative analysis of RP recordings upon elevation (**h**) or inhibition (**i**) of mimic-365. Measurements were performed on cardiac tissue acquired from 5 independent patients per each condition. Source data and detailed statistical analyses for **c**, **h**, and **i** are provided as a Source Data file.

While our experiments in human iPSC-derived cardiac myocytes demonstrate the feasibility of miR-365-based modulation of myocyte AP, such findings do not necessarily permit translation to a complex multicellular system such as the human myocardium. We, therefore, sought to manipulate miR-365 in a biomimetic culture system that allows for a consistent and stable culture of contracting human myocardium for more than 4 weeks (Fig. 7a, Supplementary Fig. 7a-c)^23^. Thin (300 µm) slices from the human left ventricular myocardium were first cultured for at least 2 weeks, during which the myocardium gradually increases contractile performance until the system equilibrated and has reached stable steady-state contraction force. By transfecting these slices, we effectively delivered miRNA mimics and antimiRs to most cells, as assessed by confocal imaging of a fluorescently labeled miRNA mimic (FAM-mimic-Ctrl, Fig. 7b). Transfection of mimic-365, resulted in a 10-fold increase of miR-365 levels in human myocardial tissue (Fig. 7c). Consequently, the expression of *KCNQ1* as the main target was reduced about 30% upon elevation of miR-365 (Supplementary Fig. 7d)

As a readout, we employed the effective refractory period (RP), defined as the interval when the tissue has not yet repolarized after a preceding excitation-contraction and is thus unresponsive to a new depolarizing stimulus (scheme in Fig. 7d). As the RP continues till the complete repolarization of the membrane, therefore, it closely correlates to AP duration^24^. In our biomimetic chamber setup, we determined the RP of the slices by measuring the contraction evoked by a programmed electrical stimulation that includes two subsequent electrical triggers set at defined and decreasing intervals (interstimulus interval). The longest interval that failed to induce two distinct contractions was considered as an approximation of the actual RP (Fig. 7d). Recordings conducted before and 48 h after transfection (Fig. 7e) revealed a significant increase of RP duration by 30 ± 5 ms upon transfection of mimic-365 (Fig. 7f, h) and a significant shortening by 50 ± 12 ms with antimiR-365 (Fig. 7g, i), compared to the respective control oligonucleotides (Supplementary Fig. 7e).

## Discussion

Here we report on miR-365, a miRNA that is highly expressed in human cardiac myocytes and that we found to regulate cardiac repolarization.

Prior to this study, relatively little has been known regarding its role in the heart. We were the first to report on a function of miR-365 in rodent cardiac myocytes, where it was a hit in a phenotypic screen for cardiomyocyte hypertrophy^25^, a finding that was confirmed and extended in another study in mice^26^. In miRNA profiling studies in various human tissue, miR-365 displayed the highest expression in skeletal muscle and myocardium, suggesting preferential expression in myocytes^27^. In human myocardium, among other miRNAs, miR-365 was suggested to have an active role in post-transcriptional repression of mRNA targets based on its incorporation in AGO2, as identified by immunoprecipitation experiments^28^. Downregulated levels of miR-365 were reported in enterovirus-induced cardiomyopathy^29^ and in heart failure patients with dilated cardiomyopathy compared to the respective controls^12,30^. MiR-365 has been found abundantly in the exosomes extracted from pericardial fluid samples from heart failure patients^31^ and as a circulatory cardiovascular-related miRNA, upregulated after exercise in human^32^.

In the present study, we manipulated miR-365 in human iPSC-derived cardiac myocytes and human myocardial slices and report a crucial role for this miRNA in the control of the human cardiac AP. Mechanistically, we identified a remarkable enrichment of canonical binding sites for miR-365 in several of the most important ion channels that determine the repolarization phase of the human cardiac AP including *KCNQ1*, *KCNH2*, *KCNJ2*, *CACNA1C*, *KCNC3*, *KCNA1*, and *KCNJ3*. While fluorescent reporter assays validated the possibility of direct interactions between miR-365 and the 3’ UTR of all of these ion channels, in-depth analysis of gene expression in cardiac myocytes at bulk and single-cell level revealed that mainly repolarizing potassium channels (namely *KCNQ1, KCNC3, KCNJ3,* and *KCNJ2*) were strongly regulated by this miRNA.

In addition to directly regulating cardiac ion channels, we found miR-365 to control important AP-regulatory factors such as potassium channel-activating kinases (*SGK3* and *SGK1*), caveolins (*CAV1* and *CAV3*), and ankyrins (*ANK2* and *ANK3*) that are shown to affect cardiac ion currents by modulating the assembly, trafficking, and activation of the ion channel proteins^33,34^. Interestingly, some of these genes are also associated with various types of LQTS (e.g., *CAV3* and *ANK2* are responsible for LQT9 and LQT4, respectively)^3^. Additionally, mRNAs coding for pore-forming or accessory domains of other repolarizing ion channels (e.g., *KCNE4*, *KCNC4*, and *KCNJ12*) were also found to be repressed upon miR-365 elevation. Thus, a picture emerges, where miR-365 orchestrates a complex set of direct and indirect targets that synergistically regulate the repolarization phase of the myocyte AP.

Electrophysiological recordings enabled us to measure such collective effects of miR-365 on key ionic currents, indicating that the concentration of miR-365 is indeed a critical determinant of the functionally dominating repolarizing current I_Ks_ (*KCNQ1*). The stoichiometry of KCNE1 to KCNQ1 expression in hiPSC-CMs is relatively lower—and the corresponding ion channel activation kinetics considerably faster—than that of primary human cardiac myocytes^35^. However, the magnitude of I_Ks_ alteration suggests that miR-365-based regulation of *KCNQ1* considerably contributes to the overall regulation of human myocyte AP duration by miR-365. In contrast, we found only subtle effects of miR-365 on the current densities of the other two prominent ion channels, that we tested, namely I_Kr_ (*KCNH2*) and I_CaL_ (*CACNA1C*), suggesting additional regulatory mechanisms that maintain the robustness of expression for these critical cardiac ion channels. Such compensatory mechanisms may depend on the cellular context, as functionally relevant targeting of *Kcnh2* by miR-365 has been reported in the central nervous system, where this interaction affected the excitability of neurons^36^.

Importantly, the complex and synergistic regulation of the AP through multiple targets in parallel may also provide for a new therapeutic opportunity. In an exemplary way, our study suggests that manipulation of miR-365 appears as an effective measure to modulate abnormal AP duration in monogenetic arrhythmias such as SQTS and LQTS. New therapeutic avenues for such forms of cardiac arrhythmia are urgently needed since current pharmacological treatments, although partially effective, met considerable challenges, that limit their applicability beyond the short-term use in acute clinical settings^37^. Likewise, more invasive options currently pursued, such as the implantation of cardioverter-defibrillators (ICDs)^4,37^ are associated with relevant device-related complications^38^.

Next to the obvious relevance for such therapeutic applications, we have designed this study on an entire human background for several reasons. First, we found the expression of miR-365 to be about 4-fold higher in human compared to mouse myocardium, which is also in good agreement with previous studies that profile human and mouse myocardium^12,28,39^. Second, recent developments in the generation of human cardiac myocytes from patient-derived iPSCs have provided human models that allow studying cardiac myocyte biology in homeostasis and disease. For our study, we exploited two models of well-defined genetic forms of QT alteration, namely LQTS and SQTS, that we have recently generated and that recapitulate the phenotype of the disease^18,19^. Naturally, limitations of hiPSC-CMs in modeling adult human cardiac myocytes apply, in particular a certain degree of immaturity^40^. Also, further analysis on hiPSC-CMs derived from larger numbers of patients LQT1 and SQT1 and other types of LQTS and SQTS will be needed to determine the broader potential of manipulating miR-365. Third, organotypic cultures of human myocardium recently saw considerable advances that now permit the culture of multiple cardiac tissue slices in parallel, with cyclic mechanical stimulation and over prolonged periods of time (up to a few months)^23^. Although these slices are prepared from failing myocardium and bear levels of functional variability (e.g., relatively prolonged RP compared to healthy myocardium), particular advantages of this model are its developmental maturity and the conservation of the native myocardial multicellularity, thus comprising also potential effects of miR-365 in non-myocytes.

Taken together, our data indicate a crucial role for miR-365 in the control of the human cardiac AP and we speculate that the application of this miRNA is not limited to channelopathies such as LQTS, but can be expanded to AP abnormalities that accompany structural heart diseases or acquired QT prolongations induced by certain drugs^41^. Considering the current advancements in oligonucleotide-based therapies in myocardium^42^, further studies to determine the therapeutic potential of miR-365 hold promise.

## Methods

### Culture, maintenance, and cardiac differentiation of hiPSCs

The family pedigrees, genetic mutations, and generation of healthy, LQT1 and SQT1 hiPSCs were described previously^16,18,19^. The somatic cells were obtained from the donors with informed consent, and the generation and characterization studies were approved by Ethics committees of Technical University of Munich, Germany (approval number 2109/08), Medical Faculty Mannheim, Heidelberg University, Germany (approval number 2009-350N-MA) and the local regulatory board (Regierung von Oberbayern, Munich, Germany). The hiPSCs were maintained in Geltrex-coated plates (1 ml per 35 mm plate, 1 h incubation at 37 °C) in E8-flex medium (2 ml per 35 mm plate, medium exchange every other day) and passaged with Versene (1 ml per 35 mm plate, incubation for 5-10 min at room temperature) when confluent. For chemically defined monolayer cardiac differentiation^17^, the cells were passaged into 12 well plates and cultured in cardio differentiation medium to reach 85–90% of confluency, the optimal cell density for starting the differentiation (d0). The differentiation carried out using Wnt signaling modulators, CHIR99021 and IWP2 (both from Merck Millipore). The yielded hiPSC-CMs were maintained in cardio culture medium and one round of metabolic selection was performed between d20-30 in order to enrich cardiomyocytes. After selection hiPSC-CMs were enzymatically dissociated (using Collagenase I and Trypsin), replated in lower densities and maintained in cardio culture medium for approximately 60 days for further maturation. Additional information about the cell lines used in this study is provided in Supplementary Data 1.

### Culture and refractory period recording in human myocardial slices

Samples of left ventricular myocardium were taken from failing hearts at the time of transplantation by the Clinic of Thoracic and Cardiovascular Surgery, Heart and Diabetes Center, Bad Oeynhausen, Germany or by the Clinic of Cardiac Surgery, University Hospital, Munich. Patients had provided informed consent to the scientific use of the explanted tissue, and the procedure has been approved by the Ethics committee of Ludwig Maximilian University of Munich (approval number 063-12). Transmural left ventricular sections were trimmed to 1 × 1 cm^2^, and were embedded in 4% low-melt agarose gel and cut into 300 µm slices using a vibratome in cold (4 °C) slicing buffer (136 mM NaCl, 5.4 mM KCl, 1 mM MgCl2, 0.33 mM NaH2PO4,10 mM glucose, 0.9 mM CaCl2, 30 mM 2,3-butadione-2-monoxime, 5 mM HEPES, pH 7.4). Slices were glued to holder triangles, mounted in biomimetic culture chambers containing 2.4 ml culture medium (Medium 199, 1% penicillin/streptomycin, 1× insulin/transferrin/selenium, and 50 µM 2-mercaptoethanol) and cultured under constant electrical stimulation (0.5 Hz, bipolar 50 mA pulse) and rocking at 60 rpm. MyoDish software (V1.1) was used for controlling these parameters and acquiring the data^23^. The slices were maintained in biomimetic chambers for at least 2 weeks before starting the experiments. The refractory period of slices was regularly recorded before and after transfection using an automated stimulation protocol consisting of a second pacing impulse at defined intervals after each regular stimulus. The intervals between both stimulations were decreased from 1000 to 250 ms in scheduled steps of 20 s duration. The longest interval that failed to induce two distinct contractions noted as the refractory period. In the end of the experiment, MakeADIbin2 was used for data conversion and the following RP analysis was performed in LabChart 8 reader (ADInstrument).

### Manipulation of miR-365 levels in human iPSC-CMs and myocardial slices

After 60 days of culture, hiPSC-CMs were replated in 96-well µplates (IBIDI) and were cultured for 7–10 days prior to the experiments to regain the contractility. To elevate the intracellular level of miR-365, the cells were transfected with synthetic mimic-365. Inhibition of endogenous miR-365 was achieved by transfection of LNA-antimiR-365 (Exiqon-Qiagen). Negative control oligonucleotides with the same chemistry were used in each case (mimic-Ctrl and antimiR-Ctrl, respectively). Transfection was performed using Lipofectamine RNAiMAX to reach the final concentration of 25 nM oligonucleotides and the effects were measured 48 h later. Myocardial slices were transfected with 1 nmol of mimic-365 or Fluorescein amidite (FAM)-labeled oligonucleotides using RNAiMax. The medium was replaced after 6–8 h. Additional information regarding the synthetic oligonucleotides is provided in Supplementary Data 2.

### Reporter activity assays

To test direct interaction between miR-365 and the target ion channels, double-fluorescent (df-) reporter constructs were cloned by insertion of 400–600 base pairs of the 3′ untranslated regions (3′ UTRs) of the ion channel genes in df-plasmids. These plasmids comprised of tdTomato gene deriving RFP expression as an internal control, and eGFP followed by the 3’ UTRs. Constructs with the mutated binding site, constructed by site-directed mutagenesis using primers carrying the mutations, served as control. The plasmids were co-transfected with mimic-Ctrl or mimic-365 (50 nM) into HEK293 cells cultured in 96-well µplate (Ibidi), using Lipofectamine2000 (Life Technologies) following the manufacturer’s recommendation.

To estimate the efficacy of miR-365 manipulation in hiPSC-CMs, cells were first transduced with an adeno-associated virus serotype 6 (AAV6) carrying a df-reporter containing three times complementary binding site for miR-365 after eGFP sequence as well as mCherry as an internal control (df-3xmiR-365). A df-reporter construct with a scrambled binding site was used as a binding specificity control. Three days after infection, miR-365 manipulation was performed as described above.

In both experiments, 48 h after transfection, the cells were fixed using 4% PFA, mounted with 50% Glycerol containing 1:100 DAPI, and GFP and RFP intensities were measured using a Zeiss Axio Observer Z1 inverted microscope. Data acquisition and analysis were performed using Metamorph software.

### Construction and production of AAV6 vectors

The plasmid containing Voltage Sensitive Fluorescent Protein-coupled to Clover GFP and mRuby2 RFP (VSFP-CR) was purchased from Addgene (#40257). The minimal promoter of *MYL2* was amplified from genomic DNA using specific primers and ligated to VSFP-CR^20^. MYL2-VSFP-CR, as well as df-3xmiR-365, were subcloned into a single-stranded AAV vector (pTREK). For packaging the AAVs, HEK293-T cells were grown in 10-layer cell stacks for 24 h, co-transfected with the desired plasmid and the helper plasmid (pDP6rs) using Polyethyleneimine (Sigma–Aldrich) and lysed after 72 h. AAVs were purified from cell lysates using an iodixanol density gradient (Optiprep, Sigma–Aldrich), concentrated in lactate and the titers (viral genome/ml) were determined by real-time PCR on viral genomes after digestion of the capsids.

### Immunostaining and confocal imaging of hiPSC-CMs and human myocardial slices

HiPSC-CMs plated in 96-well µplates (ibidi) were fixed with 4% PFA for 10 min at room temperature, and stained with primary antibodies against alpha-actinin (1:800, Sigma, A7811), ventricular myosin light chain (MYL2, 1:400, Proteintech, 10906-1-AP) for 30 min at 37 °C, followed by incubation with DAPI (1:100) and a secondary antibody (Alexa Fluor 488, goat anti-mouse IgG H + L or Alexa Fluor 594, goat anti-rabbit IgG H + L, 1:200) at 37 °C for 30 min and the stained cells were mounted with 50% glycerol. Human myocardial slices were transfected with FAM-labeled or unlabeled oligonucleotides and were embedded in OCT and snap-frozen 6–8 h after transfection. Five micrometer longitudinal section was prepared using a cryotome (Leica) and incubated with wheat germ agglutinin (WGA) labeled with Alexa Fluor-647 (1:400) for 30 min at room temperature in order to mark the plasma membrane, followed by DAPI staining (1:200) for 15 min and Sudan Black treatment to reduce autofluorescence of the tissue. Images were acquired using the confocal microscope Leica TCS SP II and the Leica Application Suite.

### Optical recording of the action potential in hiPSC-CMs

For AP recordings in whole populations of hiPSC-CMs, directly before the measurements, hiPSC-CMs were incubated with the voltage-sensitive dye di-8-ANEPPS (5 µM) at room temperature for 10 min. Pluronic F-127 (0.05%) was added to improve the solubilization of the dye. To measure the AP exclusively in ventricular hiPSC-CMs, the cells were transduced with an AAV6-MYL2-VSFP-CR^20,43^ prior to miR-365 manipulation.

For AP recording, the medium or dye solution was exchanged to pre-warmed (37 °C) FRET buffer (137 mM NaCl, 10 mM Glucose, 5.4 mM KCl, 2 mM CaCl2, 1 mM MgCl2, 10 mM HEPES, pH 7.3) and images were acquired using an inverted microscope (Zeiss AxioObserver Z1) equipped with an oil immersion ×40 objective. The cells were excited at 480 nm using a polychrome V light source (Till Photonics) and a 488 nm excitation filter and the emission spectra passed through a beam splitter (cxr565) and detected at 560 nm (Clover GFP) and 656 nm (mRuby2 RFP) using an Evolve 512 EMCCD camera (Visitron Systems). Data were acquired using MetaFluor software (Visitron Systems), preprocessed using Microsoft Excel macros and analyzed via a custom-written Python script (https://github.com/esfandyari/APDanalysis), where the action potential duration at 90% of repolarization was calculated for each cell and Bazett’s formula was used for correcting the differences in beating frequency.

### Multielectrode array measurements

For the preparation of neonatal rat cardiac myocytes 1–3-day-old pups were sacrificed and the hearts were excised and digested with collagenase type II (Worthington) and pancreatin (Sigma–Aldrich) at 37 °C. Digested cells were harvested every 10 min for 1 h, were centrifuged at 50 × *g* for 5 min, resuspended and pre-plated at 37 °C and 1% CO_2_ for 75 min onto 10 cm cell culture dishes (Nunc)^44^. The supernatant containing the cardiac myocytes was collected, and 1 million cells were plated on sterilized 16 × 16 layout MEA plate (MultiChannelSystems) coated with 5 μg/ml fibronectin in a 0.02% gelatin solution. After incubation at 37 °C overnight, the cells were transfected with 50 nM mimic-365 or control as described for hiPSC-CMs. The Field potential duration (FPD) was measured 48 h after the transfection at 37 °C using a USB-MEA256 system equipped with a temperature controller module and MC-Rack software. For MEA recordings in LQT1 and SQT1 hiPSC-CMs, 200,000 cells were seeded on each well of a ﻿60–6 well MEA 200/30 iR-Ti-tcr (MultichannelSystems) coated with Geltrex. The basal recording was performed when the cells regained spontaneous contraction (5–7 days after plating) using MEA2100-Lite-system at 37 °C. Subsequently, the cells were transfected with mimics or antimiRs and the FPD measurement was repeated after 48 h. The data were acquired using Cardio 2D software (MultichannelSystems) and Cardio 2D + was used for averaging the FPD traces and determining the FPD and the beating interval. Heart rate-corrected FPD (cFPD) values were then calculated using Bazett’s formula.

### Electrophysiology

Spontaneous action potentials in LQT1 and SQT1 hiPSC monolayers were measured 24–48 h after transfection using sharp microelectrodes for intracellular recordings. Data were acquired with a Dagan IX2-700 Dual Intracellular Pre Amp (Dagan Corporation, USA) in combination with an Axon Digidata 1440 A Low-Noise Data Acquisition System (Molecular Devices, USA). Microelectrodes were pulled with a WZ DMZ Universal microelectrode puller (Zeitz-Instruments Vertriebs GmbH, Germany) and filled with 3 M KCl. During the experiment, the cells were continuously superfused with cardio culture medium (CCM) at 35–37 °C. Recordings were taken using Clampex 10.5.2.6 software and analyzed with Clampfit 10.5.2.6 software, where the action potential duration at 90% of repolarization was calculated for each cell and Bazett’s formula was used for correcting the differences in beating frequency.

Ionic currents in LQT1 and SQT1 hiPSCs were measured 48 h after transfection with conventional patch-clamp recording techniques in the whole-cell configuration using a HEKA EPC10 USB double patch-clamp amplifier (HEKA Elektronik) and Patchmaster v2x90.2 software. Experiments were carried out at 35–37 °C, except for the I_CaL_ recordings, which were taken at room temperature. Recording electrodes were fabricated with a WZ DMZ Universal microelectrode puller (Zeitz-Instruments Vertriebs GmbH) and had resistances between 1.5 and 3.5 MΩ. To minimize the effects of the current rundown, recordings were started 3–5 min after the whole-cell configuration was established.

For I_CaL_ recordings, cells were superfused with extracellular solution consisting of 140 mM TEA-Cl, 5 mM CaCl2, 1 mM MgCl2, 10 mM HEPES, 0.01 mM TTX and 2 mM 4 AP (pH 7.4 CsOH). Pipettes were filled with intracellular solution containing: 10 mM NaCl, 135 mM CsCl, 2 mM CaCl2, 3 mM Mg-ATP, 2 mM TEA-Cl, 5 mM EGTA, and 10 mM HEPES (pH 7.2 CsOH). To measure I_CaL_ the following voltage protocol was applied: Cells were depolarized from a holding potential of −80 to −40 mV for 500 ms and then to different test potentials ranging from −100 to +60 mV in 5 mV increments for 500 ms. To obtain IV curves, the peak inward current at each test potential was determined and normalized to the cell capacitance (pA/pF). Linear leak current was subtracted.

For I_Kr_ recordings, cells were superfused with extracellular solution consisting of 130 mM NaCl, 5.4 mM KCl, 1 mM MgCl2, 1.8 mM CaCl2, 15 mM glucose, 15 mM HEPES, 1 mM Na pyruvate, 0.002 mM nifedipine, and 0.01 mM chromanol 293B (pH 7.4 NaOH). Pipettes were filled with intracellular solution containing: 125 mM K-aspartate, 5 mM KCl, 10 mM EGTA, 5 mM HEPES, 5 mM Mg-ATP, 1 mM MgCl2, 2 mM Na phosphocreatine, and 2 mM Na GTP (pH 7.2 KOH). To measure I_Kr_ the following voltage protocol was applied: Cells were depolarized from a holding potential of −40 mV to different test potentials ranging from −20 to +40 mV in 10 mV increments for 5 s. Afterward, the protocol was repeated in the presence of 0.001 mM E-4031 in the extracellular solution. I_Kr_ was calculated as the difference in current density (pA/pF) at the end of the depolarizing voltage steps. Linear leak current was subtracted.

For IKs recordings, cells were superfused with extracellular solution consisting of 130 mM NaCl, 5.4 mM KCl, 1 mM MgCl2, 1.8 mM CaCl2, 15 mM glucose, 15 mM HEPES, 1 mM Na pyruvate, 0.001 mM nifedipine, and 0.0005 mM E-4031 (pH 7.4 NaOH). Pipettes were filled with intracellular solution containing: 125 mM K-aspartate, 5 mM KCl, 10 mM EGTA, 5 mM HEPES, 5 mM Mg-ATP, 1 mM MgCl2, 2 mM Na phosphocreatine, and 2 mM Na GTP (pH 7.2 KOH). To measure I_Ks_ the following voltage protocol was applied: Cells were depolarized from a holding potential of −40 mV to different potentials ranging from −10 to +40 mV in 10 mV increments for 5 s. Afterward, the protocol was repeated in the presence of 0.01 mM chromanol 293B in the extracellular solution. I_Ks_ was calculated as the difference in current density (pA/pF) at the end of the depolarizing voltage steps. Linear leak current was subtracted.

### Quantification of miR-365 level

cDNA was prepared from 10 ng of total RNA isolated from cardiac slices using the Universal cDNA Synthesis Kit II (Exiqon). MiRCURY LNA PCR primer for miR-365 or for U6 snRNA (Exiqon) was used for qPCR on diluted cDNA (1:60) according to recommended parameters by the manufacturer.

#### RNA extraction and quantitative real-time PCR

Total RNA of neonatal rat cardiac myocytes and human cardiac slices were isolated using peqGOLD RNAPure (VWR). Slices were first homogenized using BeadBug homogenization microtubes (Sigma–Aldrich). Protoscript II Reverse Transcriptase (Invitrogen) was used cDNA synthesis and quantitative real-time PCR was performed in a StepOne Plus Real-Time-PCR System (Applied Biosystems) using FastStart universal SYBR Green Master Mix (Roche) and specific primers for each target gene (provided in Supplementary Data 3).

### Small RNA sequencing and analysis of hiPSC-CMs

Total RNA from healthy hiPSC-CMs (2 replicates) was extracted using TriFast (peqLab). The integrity and quantity of RNA were measured using Agilent Bioanalyzer 2100 and 700 ng RNA was used for library construction using NEBNext small RNA library prep kit according to manufacturer’s instructions with an AMPure XP bead-based size selection step. Libraries were sequenced on a HiSeq4000 (100 bp). Analysis was performed using an in-house Galaxy server. Forward reads were trimmed of adapters (Trim Galore! v0.4.2) and mapped against miRBase21 mature and hairpin sequences and quantified using MiRDeep2 (v2.0.0).

### RNA sequencing and analysis of hiPSC-CMs

For healthy hiPSC-CMs, the integrity and quantity of total RNA isolated from untreated and miR-365-manipulated hiPSC-CMs were measured using Agilent Bioanalyzer 2100. Libraries were prepared from 1 µg RNA using TruSeq Stranded mRNA kit (Illumina) and were sequenced on HiSeq4000 (Illumina), generating 2 × 150 bp paired-end reads. For SQT1 hiPSC-CMs, the integrity and quantity of total RNA were measured using Agilent TapeStation 4200 and Illumina mRNA ligation kit was used for library preparation, and the resulting libraries were sequenced on an SP chip of an Illumina NovaSeq6000 sequencer (2 × 100 bp, paired-end). Subsequent analysis was performed on an in-house Galaxy server. Reads were aligned against the human genome (GRCh38) using RNA STAR (v2.6.0) with default parameters. StringTie (v1.3.6) was used for transcript assembly and quantification based on GENCODE v28 (for healthy cells) or v34 (for SQT1 cells) annotations. Differentially expressed genes (>30% expression change, false discovery rate (FDR) < 0.05) amongst different treatments were verified using DESeq2 (v2.11.40.2). The biological processes associated with these genes were determined using ClueGO application (v2.5.6)^45^ ran within Cytoscape (v3.8.0)^46^ network analysis tool (*P*-value < 0.05, GO tree levels 3–8). GSEA was performed using the GSEA software (v.4.0.3, Broad Institute) on normalized reads ranked based on logFC (fold change) in mimic-365 vs mimic-Ctrl. Gene sets were extracted from the available collection in the Molecular Signatures Database (MSigDB).

### Single-cell RNA sequencing and analysis of miR-365-treated hiPSC-CMs

After transfection of hiPSC-CMs with mimics or antimiRs for 48 h, cells were digested and proceeded for single-cell capture, barcoding, and library preparation. For healthy hiPSC-CMs Chromium single-cell 3’ (v2, 10x Genomics) was used and the resulting libraries were sequenced on an Illumina HiSeq4000 to reach ~100,000 reads per cell. The reads were mapped against human genome (GRCh38, Gencode annotation v28) using CellRanger (v2.1.0, 10x Genomics). For LQT1 hiPSC-CMs, after dissociation to single cells, the cells from untreated, antimiR-Ctrl and -365- conditions were labeled using TotalSeq-A0252, 0253, and 0254 cell hashing antibodies (1 µg, Biolegend), respectively, and according to the manufacturer’s recommendations. Subsequently, the untreated, antimiR-Ctrl and antimiR-365 treated samples were pooled together at a ratio of 1:2:3 and ran through the Chromium controller. The libraries were prepared using NextGEM single-cell 3’ (v3.1, 10x Genomics) and were sequenced on an Illumina NovaSeq6000 SP chip to reach approximately 40,000 reads per cell. The samples were demultiplexed and the reads were mapped against the human genome (GRCh38, Gencode annotation v33) using CellRanger Multi (v6.0.0, 10x Genomics).

The cells that passed quality-control steps of CellRanger were subsequently analyzed using the R package Seurat (v3.2)^47^. To ensure retaining of only high-quality cells, cells with <300 genes or >6500 detected genes or >20% of mitochondrial reads were omitted from the downstream analysis. SCTransform^48^ function was used for normalization and scaling of raw counts and regressing out the unwanted sources of variation (such as mitochondrial gene content and cell cycle stage). SCTransform also yields the highly variable features that were used for linear dimensional reduction using principal component analysis (PCA). The components that contributed significantly to the dimensionality of the data were used for unsupervised graph-based clustering (resolution 0.1) and Uniform Manifold Approximation and Projection (UMAP) embedding and visualization. Adaptively thresholded Low-Rank Approximation (ALRA) algorithm was then used to impute the dropout gene expression values. The significant marker genes of each cluster were identified using Seurat FindAllMarkers function and employed to assign cell types to each cluster. Differential expression of miR-365 target ion channel genes was detected with FindMarkers function of Seurat package based on implemented Wilcoxon Rank Sum test.

### Statistical analysis

All data on bar graphs are presented as mean ± standard error of the mean (SEM). Optical and intracellular AP recording results are reported as boxplots (minimum to maximum, with median). Statistical analysis was performed using GraphPad Prism 8. Data distribution and the difference between variances were tested using the Kolmogorov–Smirnov or Shapiro-Wilk test and *F* test, respectively. Depending on the distribution of data, unpaired *t*-test or Mann–Whitney test were used to determine statistical significance. For electrophysiological measurements, statistical significance was tested using two-way ANOVA for repeated measures followed by Sidakholm post-hoc analysis. Paired *t*-test was used to evaluate the differences before and after manipulation of miR-365 in myocardial slices. Significance is depicted as *P < 0.05, **P < 0.01, ***P < 0.001, ****P < 0.0001. The detailed statistical analyses are provided as a Source Data file.

### Reporting summary

Further information on research design is available in the Nature Research Reporting Summary linked to this article.

## Supplementary information

Supplementary Information

Description of Supplementary Files

Supplementary Data 1

Supplementary Data 2

Supplementary Data 3

Reporting Summary

## Data Availability

The raw and processed RNA-sequencing data generated in this study have been deposited in the NCBI GEO database as a SuperSeries under accession number GSE185690 (including small RNA-seq under GSE185689, deep RNA-seq under GSE185687, and single-cell RNA-seq under GSE185688). The miRNA- and RNA-sequencing data used in this study^12^ are publicly available in the NCBI GEO database under accession code GSE46224. Source data for all figures are provided with this paper. All data and material generated or used in this study are available upon request. Source data are provided with this paper.

## Source data

Source data

## Code availability

The custom code for analysis of optical action potential traces is available under https://github.com/esfandyari/APDanalysis.
