# Supplementary material for: MicroRNA-365 regulates human cardiac action potential duration
Source: Nat Commun. 2022 Jan 11;13:220. doi: 10.1038/s41467-021-27856-7 (PMC8752767; doi:10.1038/s41467-021-27856-7)
Supplement: Supplementary file 1 — Supplementary Information [file 41467_2021_27856_MOESM1_ESM.pdf]

Supplementary Information

MicroRNA-365 regulates human cardiac action potential duration

Dena Esfandyari, Bio Maria Ghéo Idrissou, Konstantin Hennis, Petros Avramopoulos, Anne Dueck, Ibrahim El-Battrawy, Laurenz Grüter, Melanie Annemarie Meier, Anna Näger, Deepak Ramanujam, Tatjana Dorn, Thomas Meitinger, Christian Hagl, Hendrik Milting, Martin Borggrefe, Stefanie Fenske, Martin Biel, Andreas Dendorfer, Yassine Sassi, Alessandra Moretti, Stefan Engelhardt

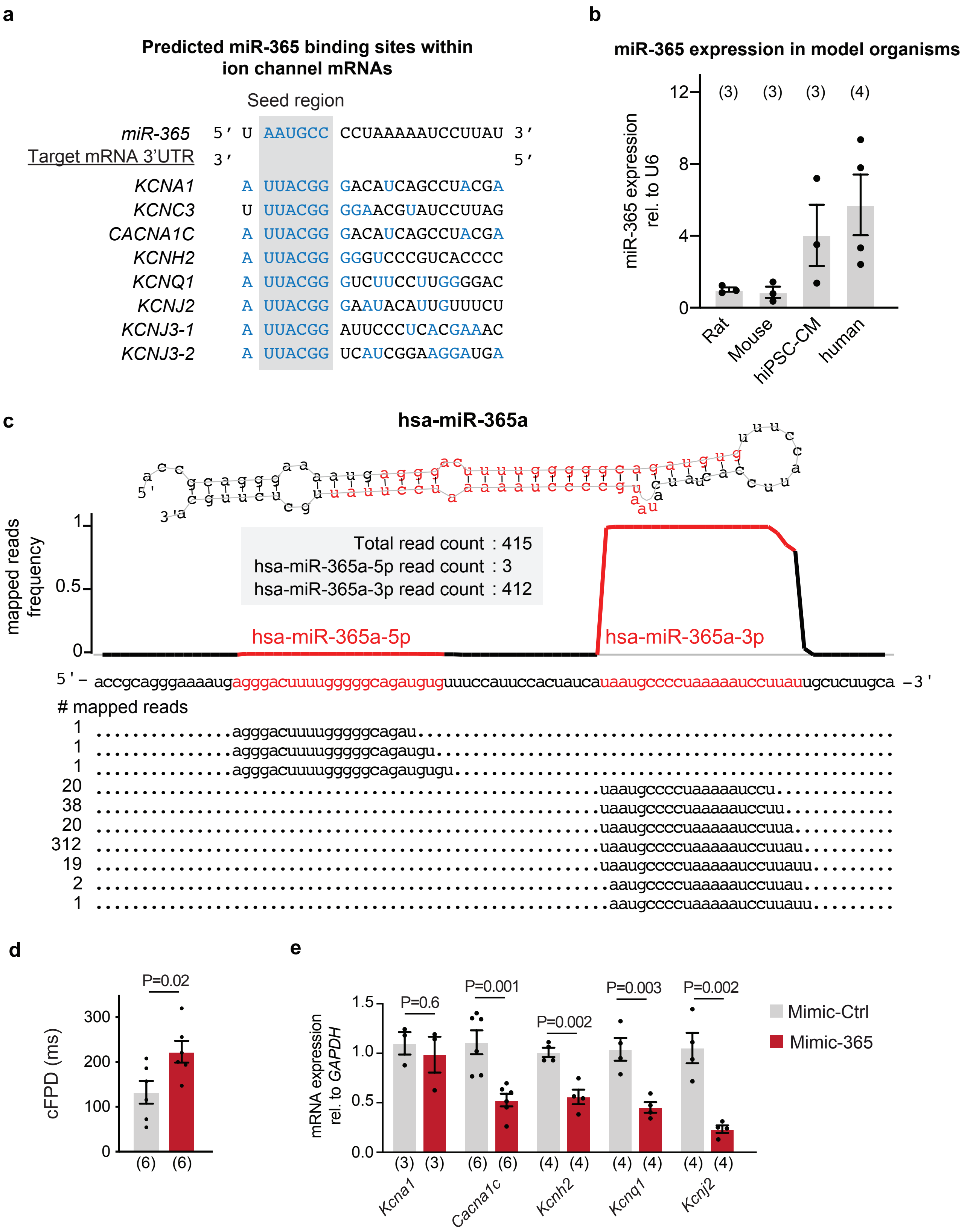

Supplementary Figure 1. MiR-365 regulates the key repolarizing potassium channels in hiPSC-CMs.
(a) Sequence of mature hsa-miR-365a and the corresponding 3' untranslated region (3' UTR) of predicted target mRNAs. The seed region (nucleotides 2-7) is highlighted in gray and the base pairing between miRNA and 3' UTRs are depicted in blue. **(b)** Relative expression of miR-365 in rat, mouse, and human myocardium and hiPSC-CMs. Data are from 3-4 independent experiments. **(c)** Proposed stem-loop structure and read signature of hsa-miR-365a precursor based on miRDeep2 analysis on small RNA-sequencing data of hiPSC-CMs. The overall frequency of the small RNA-sequencing reads and the total number of mapped reads to each part of the miRNA sequence (3p arm or mature strand and 5p arm or star strand) are depicted in the lower panels. **(d)** Multielectrode array (MEA) measurements in neonatal rat cardiac myocytes (NRCMs) shows prolongation of the beating frequency-corrected field potential duration (cFPD) upon elevation of miR-365 (n=6, each from 5-6 electrode). **(e)** Quantitative real-time PCR to assess repression of a panel of predicted targets upon elevation of miR-365. 3-6 independent experiments were performed in neonatal rat cardiac myocytes transfected with mimic-365 or mimic-Ctrl. All quantitative data are reported as means ± SEM. Source data and statistical analyses for (b), (d), and (e) are provided as a Source Data file.

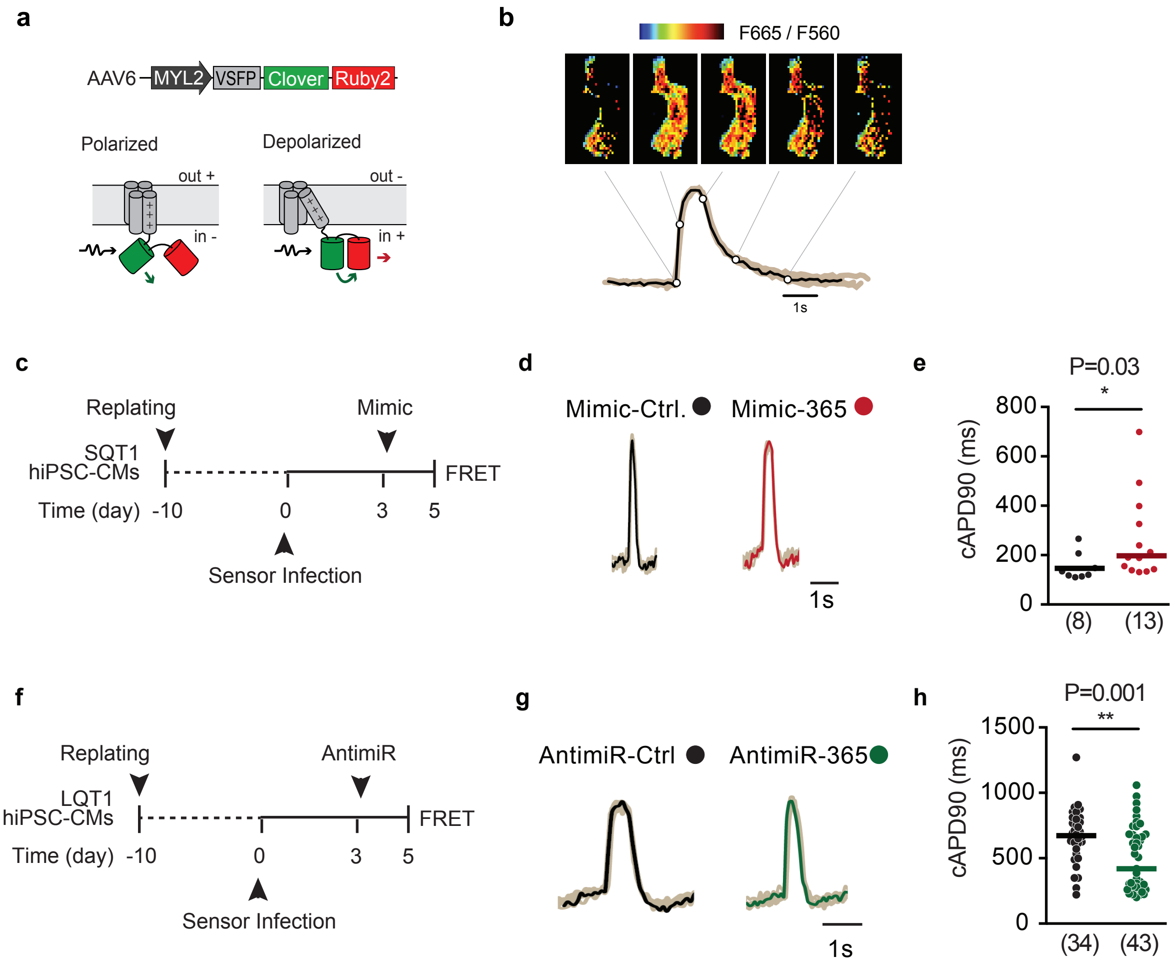

Supplementary Figure 2. FRET-based action potential recording in ventricular-like CMs. (a) Scheme showing the adeno-associated virus (AAV, serotype 6) carrying a genetically encoded, FRET-based voltage sensor (VSFP-CR) under control of the ventricular-specific MYL2 promoter. **(b)** Average trace of 15 APs recorded from a representative cell transduced with AAV6-MYL2-VSFP. Energy transfer between the two fluorophores is depicted as ratio of Ruby2-RFP to Clover-GFP emissions (F665/F560) in specific time-points of the AP (white circles). **(c)** Experimental scheme representing FRET-based AP recording in SQT1 hiPSC-CMs. The hiPSC-CMs were transduced with AAV6-MYL2-VSFP-CR and after robust expression of the sensor, the cells were transfected with mimic-365 or -Ctrl for 48 hours. **(d)** Averaged AP traces (10-15 single APs) recorded from representative SQT1 hiPSC-CMs treated with mimic-Ctrl (black) or mimic-365 (red). **(e)** Quantification of optical AP recordings selectively in ventricular-like SQT1 myocytes using MYL2-VSFP-CR (median, depicted by lines, are 127 ms and 190 ms in the mimic-Ctrl and -365 treated cells, respectively). Data are acquired from 2 independent experiments. **(f)** Experimental scheme of FRET-based AP recording in LQT1 hiPSC-CMs after miR-365 inhibition. **(g)** Averaged AP traces (10-15 single APs) recorded from representative LQT1 hiPSC-CMs treated with antimiR-Ctrl (black) or antimiR-365 (green). **(h)** Quantification of optical AP recordings selectively in ventricular-like LQT1 hiPSC-CMs using MYL2-VSFP-CR (median 673 ms in the antimiR-Ctrl group compared to 418 ms in antimiR-365 treated cells). Data acquired from 4 independent experiments. Source data and statistical analyses for (e) and (h) are provided as a Source Data file.

a

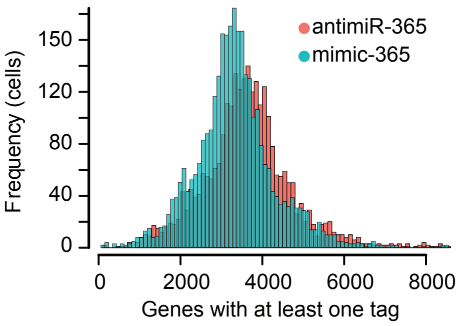

b

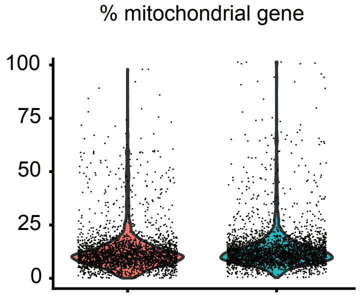

c

	Mimic-365 treated hiPSC-CMs	AntimiR-365 treated hiPSC-CMs
**Cells and detected genes**		
Estimated Number of Cells	3,255	2,724
Fraction Reads in Cells	87.20%	85.50%
Mean Reads per Cell	114,416	133,849
Median Genes per Cell	3,437	3,610
Total Genes Detected	25,774	25,932
Median UMI Counts per Cell	15,527	16,853
Number of Reads	372,425,335	364,606,792
Valid Barcodes	97.70%	97.80%
Sequencing Saturation	68.40%	70.60%
**Mapping**		
Reads Mapped to Genome	87.70%	86.30%
Reads Mapped Confidently to Genome	83.50%	82.60%
Reads Mapped Confidently to Intergenic Regions	3.40%	3.00%
Reads Mapped Confidently to Intronic Regions	18.30%	18.00%
Reads Mapped Confidently to Exonic Regions	61.80%	61.50%
Reads Mapped Confidently to Transcriptome	57.20%	57.30%
Reads Mapped Antisense to Gene	0.90%	0.90%

d

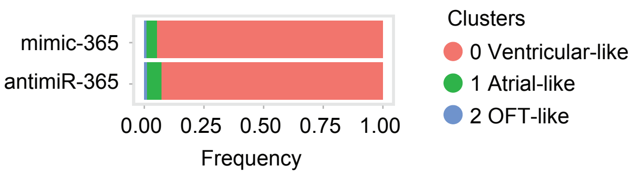

Supplementary Figure 3. Single cell RNA-sequencing data quality metrics for hiPSC-CMs treated with antimiR- or mimic-365. (a) Histogram depicting the distribution of number of detected genes per cell in healthy hiPSC-CMs treated with antimiR- (red) or mimic-365 (blue). **(b)** Violin plots depicting the percentage of reads corresponding to mitochondrial gene content in hiPSC-CMs treated with antimiR- or mimic-365. These parameters were used for preprocessing and quality control in Seurat R package. **(c)** Table depicting scRNA-seq data quality metrics and read mapping efficiency. **(d)** Abundance of each cell type in healthy hiPSC-CM population treated with antimiR- or mimic-365.

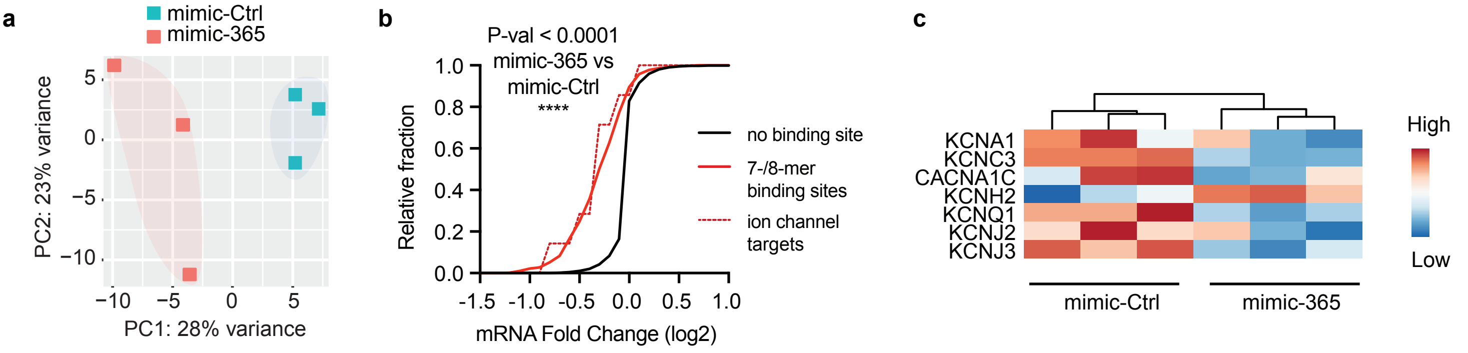

Supplementary Figure 4. Transcriptome analysis of STQ1 hiPSC-CMs manipulated with miR-365 mimics. (a) Principal component analysis (PCA) of mimic-Ctrl- and mimic-365- treated SQT1 hiPSC-CMs. **(b)** Cumulative distribution plots of differential expression for all genes versus broadly conserved miR-365 targets containing 8-mer, 7-mer-m8, or 7-mer-A1 binding sites as predicted by TargetScan Human version 7.2. Kolmogorov-Smirnov test was used to determine the significance (P-value < 0.0001). **(c)** Heatmap depicting the expression of ion channel targets of miR-365 upon overexpression of this miRNA in SQT1 hiPSC-CMs.

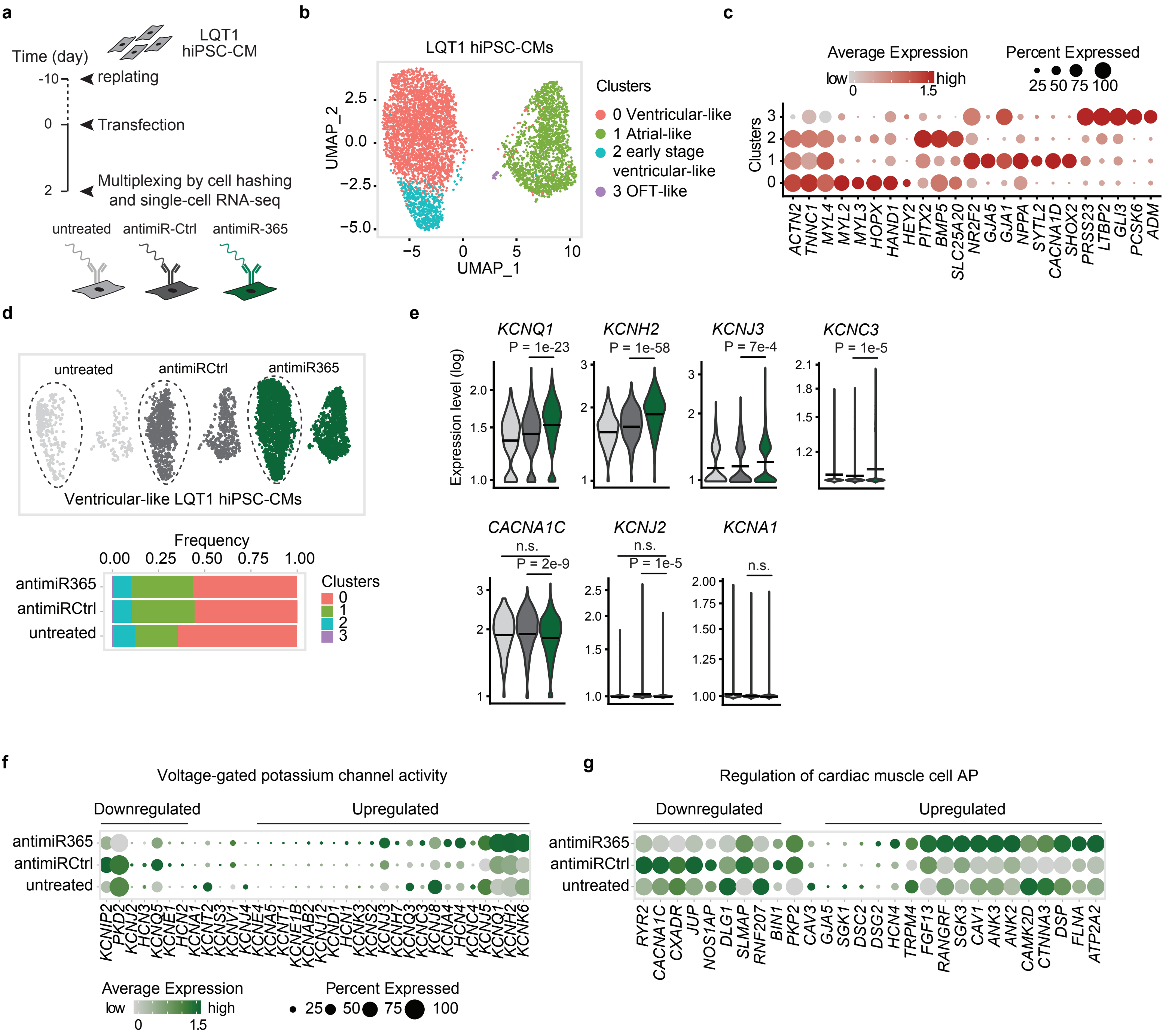

Supplementary Figure 5. Regulation of the key repolarizing potassium channels in LQT1 cardiac myocytes. (a) Schematic overview of the single cell RNA sequencing experiment in patient-specific LQT1 hiPSC-CMs. The untreated cell and cells transfected with antimiR-365 or -Ctrl were isolated, labeled with cell hashing barcode-conjugated antibodies and were multiplexed prior to droplet-based single-cell RNA-seq. **(b)** Uniform manifold approximation and projection (UMAP) plot representing the unbiased clustering of 8000 transcriptomes revealed four main populations of cells, namely early and late-stage ventricular-like, atrial-like and other cardiac myocytes. **(c)** Dot plot showing the expression of marker genes defining each subpopulation of hiPSC-CMs. **(d)** UMAP clustering of LQT1 hiPSC-CM split based on the treatment group (upper panel) and the contribution of cells from each sample to different hiPSC-CM clusters (lower panel). **(e)** Violin plots representing the expression changes of ion channel targets of miR-365 in ventricular-like cardiac myocytes upon inhibition of endogenous miR-365. Analyses were performed using CellRanger and Seurat (v3) packages. Adjusted P-value was calculated using Wilcoxon Rank Sum test for each gene. **(f, g)** The abundance of genes involved in GO terms “voltage gated potassium channel activity” (f) and “regulation of cardiac muscle cell action potential” (g) at single cell level in LQT1 hiPSC-CMs upon treatment with antimiRs.

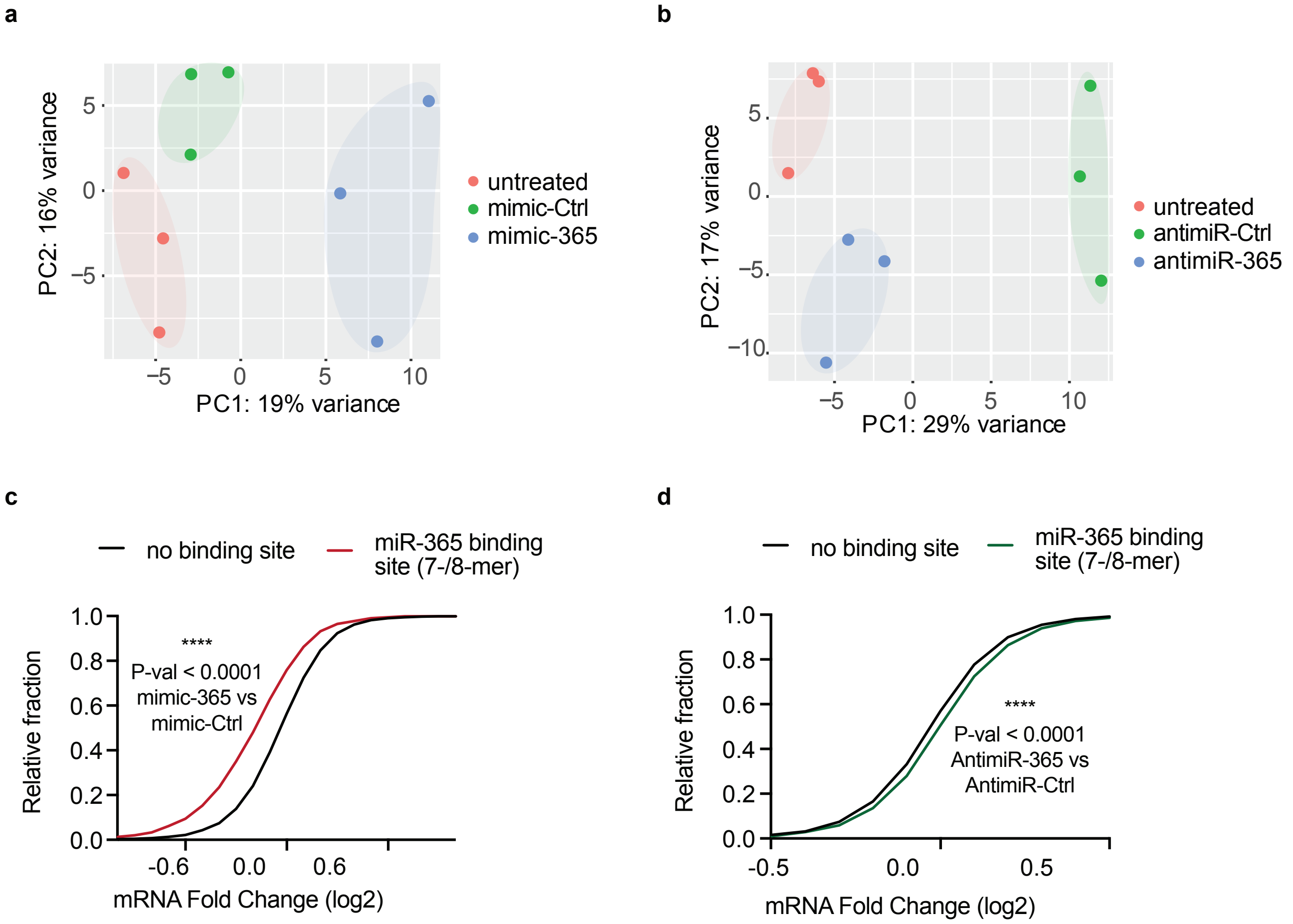

Supplementary Figure 6. Transcriptome analysis of hiPSC-CMs manipulated with miR-365 mimic and antimiR. (a) Principal component analysis (PCA) of untreated, mimic-Ctrl- and mimic-365- treated hiPSC-CMs. **(b)** Cumulative distribution plots of differential expression for all genes versus broadly conserved miR-365 targets containing 8-mer, 7-mer-m8, or 7-mer-A1 binding sites as predicted by TargetScan Human version 7.2. Kolmogorov-Smirnov test was used to determine the significance (P-value < 0.0001). **(c)** Principal component analysis of untreated, antimiR-Ctrl- and antimiR-365- treated hiPSC-CMs. **(d)** Cumulative distribution plots of expression changes for all genes versus broadly conserved miR-365 targets carrying 8-mer, 7-mer-m8, or 7-mer-A1 binding sites (predicted by TargetScan Human version 7.2). Kolmogorov-Smirnov test was used to determine the significance (**** P-value < 0.0001).

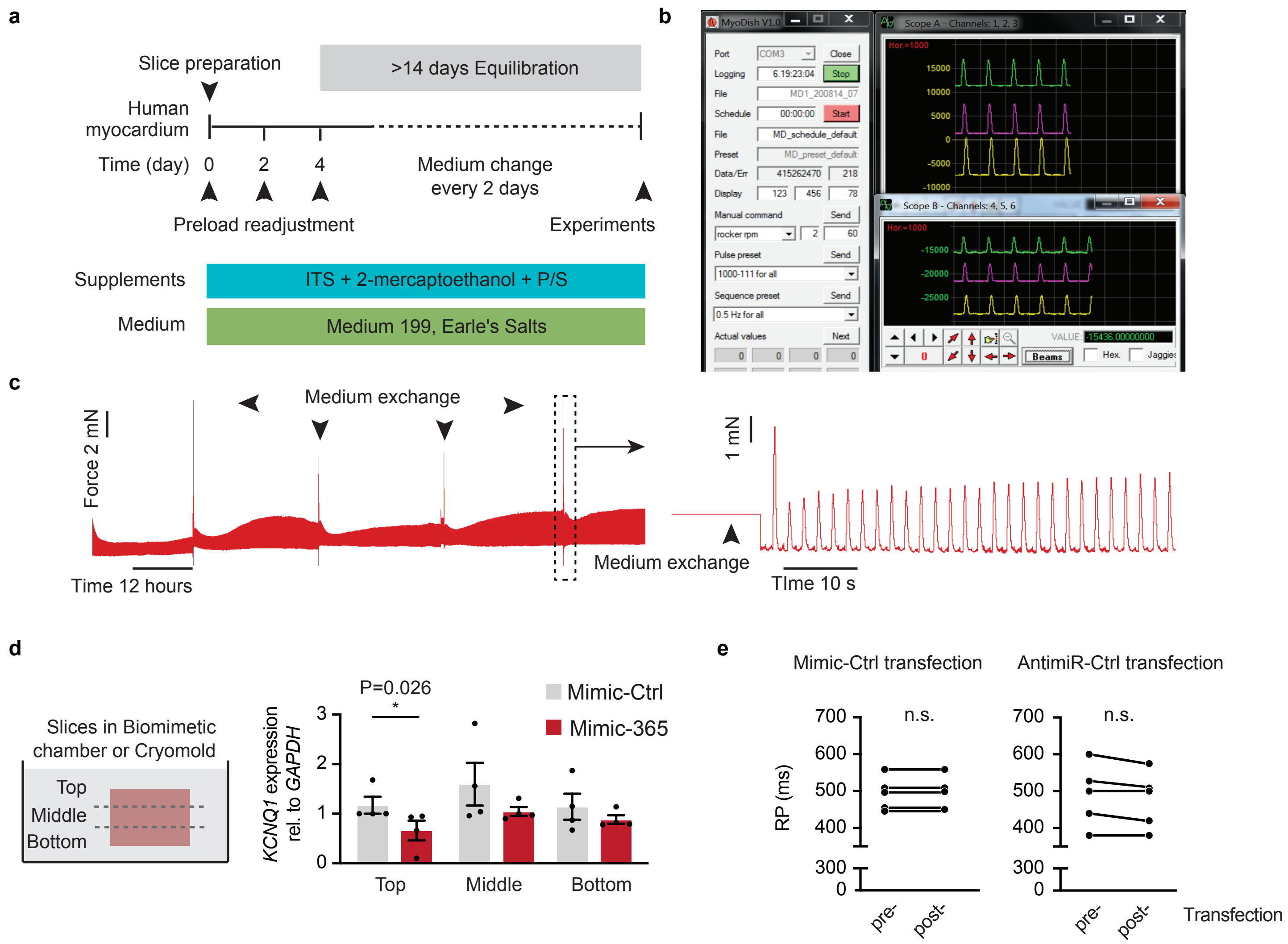

Supplementary Figure 7. Human myocardial slices as an ex vivo model to study the role of miR-365 in AP regulation. (a) Scheme showing the steps in preparation and cultivation of human myocardial slices. **(b)** Screenshot of the data acquisition software (Myodish v1.0) representing the controller options (right) and exemplary contraction force traces from 6 different slices (right). **(c)** Contraction force measurement from a representative slice over a 10-day time span of continuous cultivation (left) and at a higher resolution immediately after the medium exchange (right). **(d)** Scheme representing the localization of human myocardial slices inside the biomimetic culture dish or embedded in a cryomold before making sections for qPCR (left) and qPCR results for KCNQ1, a validated target of miR-365, in different layers of the slices. Data is acquired from 4 independent myocardial samples. All quantitative data are reported as means ± SEM. **(e)** Quantitative analysis of RP recordings upon transfection of myocardial slices with control oligonucleotides (mimic-Ctrl and antimiR-Ctrl). Measurements were performed on cardiac tissue acquired from 5 independent patients per each condition (n.s., non-significant based on paired t-test). Source data and statistical analyses for (d) and (e) are provided as a Source Data file.
