# Supplementary material for: MicroRNA-365 regulates human cardiac action potential duration
Source: Nat Commun. 2022 Jan 11;13:220. doi: 10.1038/s41467-021-27856-7 (PMC8752767; doi:10.1038/s41467-021-27856-7)
Supplement: Supplementary file 2 — Description of Supplementary Files [file 41467_2021_27856_MOESM2_ESM.docx]

**MicroRNA-365 regulates human cardiac action potential duration**

Dena Esfandyari, Bio Maria Ghéo Idrissou, Konstantin Hennis, Petros Avramopoulos, Anne Dueck, Ibrahim El-Battrawy, Laurenz Grüter, Melanie Annemarie Meier, Anna Näger, Deepak Ramanujam, Tatjana Dorn, Thomas Meitinger, Christian Hagl, Hendrik Milting, Martin Borggrefe, Stefanie Fenske, Martin Biel, Andreas Dendorfer, Yassine Sassi, Alessandra Moretti, Stefan Engelhardt

**Description of Additional Supplementary Files:**

**File Name: Supplementary Data 1**.

**Description:** Cell lines used in the study.

**File Name: Supplementary Data 2.**

**Description:** Oligonucleotide sequences used in the study.

**File Name: Supplementary Data 3.**

**Description:** Primer sequences used for qPCR in the study.
