# Supplementary material for: MicroRNA-365 regulates human cardiac action potential duration
Source: Nat Commun. 2022 Jan 11;13:220. doi: 10.1038/s41467-021-27856-7 (PMC8752767; doi:10.1038/s41467-021-27856-7)
Supplement: Supplementary file 6 — Reporting Summary [file 41467_2021_27856_MOESM6_ESM.pdf]

nature research

Corresponding author(s): Stefan Engelhardt

Last updated by author(s): Nov 15, 2021

Reporting Summary

Nature Research wishes to improve the reproducibility of the work that we publish. This form provides structure for consistency and transparency in reporting. For further information on Nature Research policies, see our Editorial Policies and the Editorial Policy Checklist.

Statistics

For all statistical analyses, confirm that the following items are present in the figure legend, table legend, main text, or Methods section.

n/a	Confirmed	
☐	☒	The exact sample size (*n*) for each experimental group/condition, given as a discrete number and unit of measurement
☐	☒	A statement on whether measurements were taken from distinct samples or whether the same sample was measured repeatedly
☐	☒	The statistical test(s) used AND whether they are one- or two-sided<br>*Only common tests should be described solely by name; describe more complex techniques in the Methods section.*
☒	☐	A description of all covariates tested
☐	☒	A description of any assumptions or corrections, such as tests of normality and adjustment for multiple comparisons
☐	☒	A full description of the statistical parameters including central tendency (e.g. means) or other basic estimates (e.g. regression coefficient) AND variation (e.g. standard deviation) or associated estimates of uncertainty (e.g. confidence intervals)
☐	☒	For null hypothesis testing, the test statistic (e.g. *F*, *t*, *r*) with confidence intervals, effect sizes, degrees of freedom and *P* value noted<br>*Give P values as exact values whenever suitable.*
☒	☐	For Bayesian analysis, information on the choice of priors and Markov chain Monte Carlo settings
☒	☐	For hierarchical and complex designs, identification of the appropriate level for tests and full reporting of outcomes
☒	☐	Estimates of effect sizes (e.g. Cohen's *d*, Pearson's *r*), indicating how they were calculated

Our web collection on statistics for biologists contains articles on many of the points above.

Software and code

Policy information about availability of computer code

Data collection	commercial data acquisition tools:<br>microscopy:<br>Leica Application Suite Advanced Fluorescence, version 2.7.9723<br>Metafluor imaging, version 7.6.5.0<br>MetaMorph Premier imaging system, version 7.10.1<br>electrophysiology:<br>Cardio2D MultichannelSystems, version 2.14.2<br>Patchmaster, version 2x90.2<br>Clampex, version 10.5.2.6
Data analysis	CellRanger, version 2.1.0 and 6.0.0<br>Seurat, version 3.2<br>Trim Galore!, version 0.4.2<br>miRDeep2, version 2.0.0<br>STAR, version 2.6.0<br>StringTie, version 1.3.6<br>DESeq2, version 2.11.40.2<br>Cytoscape, version 3.8.0<br>ClueGO, version 2.5.6<br>GSEA 4.0.3<br>MyoDish software version 1.1<br>MetaMorph Offline, version 7.10.1

Microsoft Excel for Mac version 15.51
Python-based APD analysis script (https://github.com/esfandyari/APDanalysis)
Cardio2D MultichannelSystems, version 2.9.2
MC_Rack MultichannelSystems, version 4.6.2
Clampfit, version 10.5.2.6
Prism 8 for Mac OS, version 8.4.1

For manuscripts utilizing custom algorithms or software that are central to the research but not yet described in published literature, software must be made available to editors and reviewers. We strongly encourage code deposition in a community repository (e.g. GitHub). See the Nature Research guidelines for submitting code & software for further information.

Data

Policy information about availability of data

All manuscripts must include a data availability statement. This statement should provide the following information, where applicable:

- Accession codes, unique identifiers, or web links for publicly available datasets
- A list of figures that have associated raw data
- A description of any restrictions on data availability

The raw and processed RNA-sequencing data generated in this study have been deposited in the NCBI GEO database as a SuperSeries under accession number GSE185690 [https://www.ncbi.nlm.nih.gov/geo/query/acc.cgi?acc=GSE185690](including small RNA-seq under GSE185689 [https://www.ncbi.nlm.nih.gov/geo/query/acc.cgi?acc=GSE185689], deep RNA-seq under GSE185687 [https://www.ncbi.nlm.nih.gov/geo/query/acc.cgi?acc=GSE185687] and single cell RNA-seq under GSE185688 [https://www.ncbi.nlm.nih.gov/geo/query/acc.cgi?acc=GSE185688]). The miRNA- and RNA-sequencing data used in this study are available in the NCBI GEO database under accession code GSE46224 [https://www.ncbi.nlm.nih.gov/geo/query/acc.cgi?acc=GSE46224]. Source data for all figures are provided with this paper. All data and material generated or used in this study are available upon request.

Field-specific reporting

Please select the one below that is the best fit for your research. If you are not sure, read the appropriate sections before making your selection.

☒ Life sciences ☐ Behavioural & social sciences ☐ Ecological, evolutionary & environmental sciences

For a reference copy of the document with all sections, see nature.com/documents/nr-reporting-summary-flat.pdf

Life sciences study design

All studies must disclose on these points even when the disclosure is negative.

Sample size	The exact sample size for each experiment is provided in the figures, legends and the text.<br>No prior calculation was performed to determine the sample size, however, the sample size used in each experiment was sufficiently large to observe the impact of miR-365 on AP duration and to perform statistical analysis, as further validated by multiple replications.<br>For human myocardial slices (n=5), the sample size was determined based on availability of myocardial material. For AP recordings in hiPSC-CMs, the sample size was comparable to other studies with similar design (n=18-80 for optical recordings, similar to Chen Z., EHJ, 2016, Fig.3, n=8-18 cells for patch-clamp experiments, similar to Verkerk A, Int J Mol Sci, 2017, Table 1). For double-fluorescent reporter assays more than 10,000 single cells were analyzed by high-content microscopy.
Data exclusions	No data were excluded from the analysis.
Replication	All functional AP recordings in hiPSC-CMs were collected from more than 3 independent experiments. For double-fluorescent experiments 4 images per well were acquired from 2 wells per condition in each experiment and at least 3 independent experiments were performed. 3 replicates per condition were included for the RNA-seq experiments. For single cell RNA-seq experiments, the transcriptome of more that 14,000 cells was analyzed. Data on myocardial slices were acquired from 5 independent patients. All attempts in replication were successful.
Randomization	The cells and myocardial slices were assigned to experimental and control groups randomly.
Blinding	Treatments of the cells and myocardial slices were performed by only one investigator and therefore blinding was not possible, however, the transfection protocol (amount of oligonucleotides, incubation time, amount of reagents added to the samples) was exactly identical for miR-365-specific and control oligonucleotides, excluding the possibility of any bias. Additionally, the acquisition and analysis of optical AP recordings, high content microscopy for double-fluorescent reporter assays and RP recordings in slices were done in a batch and the investigators were not directly aware of the treatment conditions.

Reporting for specific materials, systems and methods

We require information from authors about some types of materials, experimental systems and methods used in many studies. Here, indicate whether each material, system or method listed is relevant to your study. If you are not sure if a list item applies to your research, read the appropriate section before selecting a response.

Materials & experimental systems

n/a	Involved in the study
☐	☒ Antibodies
☐	☒ Eukaryotic cell lines
☒	☐ Palaeontology and archaeology
☒	☐ Animals and other organisms
☐	☒ Human research participants
☒	☐ Clinical data
☒	☐ Dual use research of concern

Methods

n/a	Involved in the study
☒	☐ ChIP-seq
☒	☐ Flow cytometry
☒	☐ MRI-based neuroimaging

Antibodies

Antibodies used

Mouse monoclonal Anti-α-Actinin (Sarcomeric, Sigma Aldrich A7811, clone EA-53)
Ventricular myosin Light Chain 2 (MYL2), Rabbit polyclonal (Proteintech 10906-1-AP)
wheat germ agglutinin (WGA), Alexa Fluor-647 conjugate (Invitrogen W32466)
Alexa Fluor 488, goat anti-mouse IgG H+L (Thermo Fisher A28175)
Alexa Fluor 594, goat anti-rabbit IgG H+L (Thermo Fisher A11037)
TotalSeq™-A0252 anti-human Hashtag 2 Antibody (Biolegend 394603)
TotalSeq™-A0253 anti-human Hashtag 3 Antibody (Biolegend 394605)
TotalSeq™-A0254 anti-human Hashtag 4 Antibody (Biolegend 394607)

Validation

All the primary antibodies were purchased from companies and used according to manufacturer's recommendations. Anti-α-Actinin and MYL2 antibodies were tested for IF in human and mouse heart by the manufacturers as well as by extensive use in published works (https://www.sigmaaldrich.com/DE/de/product/sigma/a7811, https://www.ptglab.com/Products/Pictures/pdf/10906-1-AP.pdf). TotalSeq antibodies are tested by the company by PCR and sequencing to confirm the oligonucleotide barcodes and by flow cytometry to ensure the antibodies recognize the proper cell populations (https://www.biolegend.com/en-us/products/totalseq-a0254-anti-human-hashtag-4-antibody-16086?GroupID=GROUP28).

Eukaryotic cell lines

Policy information about cell lines

Cell line source(s)

HEK293T, HEK293 (ATCC)
hiPS cell lines from control, long QT and short QT syndrome patients described before in (Gramlich, EMBO Mol. Med. 2015, Moretti, NEJM 2010, El-battrawy, JAHA 2018).
NRCMs prepared in this work.
More information can be found in Supplementary Table 1.

Authentication

All the hiPSCs were validated in the original studies (Gramlich, EMBO Mol. Med. 2015, Moretti, NEJM 2010, El-battrawy, JAHA 2018) by various means such as staining with pluripotency markers (NANOG, SOX2) and determining differentiation capacity. HEK293 and HEK293T cell lines were purchased from commercial vendors where STR profiling was performed. No additional authentication was performed.

Mycoplasma contamination

All cell lines were regularly tested for mycoplasma contamination and no contaminations were detected.

Commonly misidentified lines
(See ICLAC register)

None is used.

Human research participants

Policy information about studies involving human research participants

Population characteristics	Donors of somatic cells for generation of healthy, LQT1 and SQT1 hiPS cells were a 62-year-old female, a 42-year-old male, and a 29-year-old male, respectively (Gramlich, EMBO Mol. Med. 2015, Moretti, NEJM 2010, El-battrawy, JAHA 2018).<br>For preparation of slices, 6 male patients (age: 27-60 years old) receiving a heart transplantation at the Clinic of Thoracic and Cardiovascular Surgery, Heart and Diabetes Center, Bad Oeynhausen, Germany or at he Clinic of Cardiac Surgery, University Hospital, Munich, Germany were involved in the study.
Recruitment	The patients were in the cardiac transplantation list at the Clinic of Thoracic and Cardiovascular Surgery, Heart and Diabetes Center, Bad Oeynhausen, Germany or by the Clinic of Cardiac Surgery, University Hospital, Munich, Germany, and there were no self-selection involved. Patients had provided informed consent to the scientific use of the explanted tissue. Samples of left ventricular myocardium were taken from failing hearts at the time of transplantation.<br>Recruitment of the donors for generation of somatic cells was described in the respective publications (Gramlich, EMBO Mol. Med. 2015, Moretti, NEJM 2010, El-battrawy, JAHA 2018).
Ethics oversight	The somatic cells were obtained from the donors with informed consent, and the generation and characterization studies were approved by Ethics committees of Technical University of Munich, Germany (approval number 2109/08), Medical Faculty Mannheim, Heidelberg University, Germany (approval number 2009-350N-MA) and the local regulatory board (Regierung von Oberbayern, Munich, Germany). The cardiac slice experiments have been approved by the Ethics committee of Ludwig Maximilian University of Munich (approval number 063-12).

Note that full information on the approval of the study protocol must also be provided in the manuscript.
